# TRK-T3 condensate organization drives growth signaling

**DOI:** 10.26508/lsa.202603653

**Published:** 2026-06-17

**Authors:** Sindy R Chavez, Savannah M Bogus, Samantha N Cheung, Winnie Zhou, Lilja Gudmundsson, Kaho Kato, Andreas M Ernst

**Affiliations:** Department of Cell and Developmental Biology, School of Biological Sciences, University of California San Diego, La Jolla, CA, USA

## Abstract

Oncogenic fusion proteins involving receptor tyrosine kinases can hijack cell signaling cascades to promote oncogenesis. Here, we show that the TRK-T3 fusion protein, generated by joining the N-terminal region of the condensate-forming protein TFG with the C-terminal region of TrkA, assembles into sheet-like biomolecular condensates after putative prewetting of the ER. These structures are distinct from those formed by either parent protein. We find that ER wetting and the condensate morphology of TRK-T3 are controlled by a hydrophobic transmembrane segment encoded in the TrkA moiety. Removing this segment abolishes the sheet-like morphology of TRK-T3 condensates in cells and in vitro. This loss of morphology diminishes the recruitment of the first-layer signal-transduction protein GRB2 and suppresses activation of the MAPK pathway. A minimal segment that mimics the transmembrane domain efficiently disrupts TRK-T3 condensation. Together, our findings highlight condensate morphology as an encoded property of this oncogenic fusion protein, controlling the alignment and signaling competence of TRK-T3 assemblies. They further suggest that hydrophobic interactions within TRK-T3 condensates represent a potential vulnerability in this oncogenic signaling mechanism.

## Abstract (precis)

The TRK-T3 oncogenic fusion protein forms biomolecular condensates driven by a hydrophobic segment that drives both morphology and MAPK activation.

## Introduction

Chromosomal rearrangements may generate oncogenic fusion proteins that involve receptor tyrosine kinases (RTKs), which can promote oncogenesis by creating artificial proliferative signaling cues. These fusion proteins often drive a constitutive activation of pathways involved in proliferation, which can lead to genome destabilization (Negrini et al, 2010; Fernandez-Medarde & Santos, 2011; Gao et al, 2018). The RTK/Ras GTPase/MAP kinase (MAPK) pathway can be hyperactivated by fusion proteins that create novel functional proteins capable of inducing growth signaling in the absence of growth factors. Growth signaling is frequently mediated by kinase domains within fusions that stem from a RTK protein (Mitelman et al, 2007; Cuneo & Mittag, 2021). Frequently, these kinase domains can become fused to fragments of unrelated proteins that provide oligomerization or scaffolding domains. These fusions can cause kinase domains to become autophosphorylated, resulting in both their constitutive phosphorylation and activation of signaling pathways via recruitment of signal-transduction molecules (Mitelman et al, 2004; Cuneo & Mittag, 2021). Recent studies have suggested that several oncogenic fusion proteins can form biomolecular condensates that concentrate factors such as GRB2 and SOS1, supporting a model in which an altered intracellular distribution and properties of the condensate dense phase can significantly impact initiation of oncogenic MAPK signaling (Tulpule et al, 2021; Shirnekhi et al, 2023; Zhu et al, 2023).

TRK-T3 is an oncogenic fusion protein that combines the housekeeping protein Trk-fused gene (TFG) with a neuronal-specific RTK protein, tropomyosin receptor kinase A (TrkA). The TRK-T3 fusion is formed from the amino-terminal (N-terminal) amino acids 1–193 of the protein TFG and the carboxy-terminal (C-terminal) amino acids 399–796 of the protein TrkA. The TFG open reading frame was originally identified through its fusion to TrkA (Greco et al, 1995) and is now established to contribute to the organization of the early secretory pathway, particularly at the interface between the ER and the Golgi apparatus (Witte et al, 2011; Hanna et al, 2017; Qiu et al, 2024; Bogus et al, 2025). Although largely intrinsically disordered, TFG contains three folded domains that are retained in TRK-T3, including a Phox and Bem1 (PB1) domain that contributes to homo-oligomerization (Witte et al, 2011), and two alpha-helical domains (Bogus et al, 2025). TFG, a phosphoprotein, was shown to undergo a liquid–liquid phase separation (LLPS) to form anisotropic condensates at ER exit sites resembling hollow spheres, which structure the ER–Golgi interface and are proposed to organize bidirectional traffic within it (Bogus et al, 2025). The specific condensate geometry of TFG was shown to involve a molecular condensation mechanism based on hydrophobic “sticker” residues within its intrinsically disordered moiety (Bogus et al, 2025). The N-terminal region of TFG, which is present in TRK-T3, interacts with the peripheral ER exit site (ERES) protein Sec16 (Witte et al, 2011). The TrkA portion of the fusion contains both the TrkA transmembrane and a kinase domain, although the absence of an amino-terminal signal sequence in the fusion protein prevents TRK-T3 from becoming co-translationally inserted into the ER membrane, thus resulting in a cytosolic fusion protein, which raises the question of how the TRK-T3 fusion can activate the MAPK pathway from within the cytosol.

TRK-T3 has been shown to directly trigger oncogenesis. It can induce uncontrolled proliferation when expressed in NIH/3T3 cells, drive tumor formation in mice, and activate MAPK signaling (Greco et al, 1995; Edel et al, 2004; Witte et al, 2011). A coiled coil domain encoded in the TFG moiety was reported to impact the extent of cell transformation (Greco et al, 1998). An alternatively spliced variant of TRK-T3 has been reported to form condensates, although this isoform lacks the transmembrane domain (TMD) present in the originally identified fusion (Greco et al, 1995; Zhu et al, 2023). Previous research proposed that the TFG moiety encoded in TRK-T3 mediates its localization to ERES, creating a feedforward mechanism of phosphorylation and MAPK activation (Witte et al, 2011). This leaves open the question as to whether the TFG moiety solely functions as an ERES targeting signal, or whether its propensity to form anisotropic condensates could contribute to kinase domain alignment, autophosphorylation, and recruitment of signal-transduction molecules.

Here, we investigated whether LLPS of TRK-T3 could contribute to the molecular mechanism of cell transformation. Unexpectedly, using cell-based and in vitro assays, we find that it forms sheet-like biomolecular condensates that enrich in the vicinity of the plasma membrane (PM). We find that this unusual condensate morphology is mediated by a hydrophobic segment within the TrkA moiety, the TMD. Removing this segment results in a loss of condensate morphology, as well as both the ability to recruit the signal-transduction molecule GRB2 and activate the MAPK pathway. Furthermore, we show that in the presence of a hydrophobic minimal sequence that mimics the TrkA-TMD, TRK-T3 is no longer able to form condensates, both in cells and in vitro. We propose a model wherein the hydrophobic segment drives a putative planar condensate geometry to align TRK-T3 and its kinase domains, which results in efficient autophosphorylation and recruitment of signal-transduction molecules. Here, flattened condensates mimic the alignment of native RTKs in the PM and their ligand-induced oligomerization, thus efficiently bypassing control via external stimuli and resulting in aberrant MAPK activation and oncogenesis.

## Results

### TRK-T3 assembles into sheet-like condensates in cells

We previously reported that the protein TFG forms anisotropic biomolecular condensates at ERES that may serve to control bidirectional traffic between the ER and Golgi (Bogus et al, 2025). TFG has been found to form oncogenic fusion proteins with several RTKs, including RET, ALK, FGFR, and TrkA (Greco et al, 1995; Hernández et al, 1999; Loong et al, 2020; Wang et al, 2020). Recently, it has been shown that a subset of oncogenic fusion proteins can form biomolecular condensates through LLPS, which may contribute to cell transformation if the resulting condensates sequester signal-transduction molecules involved in proliferative signaling (Tulpule et al, 2021; Zhu et al, 2023).

**Figure 1.**
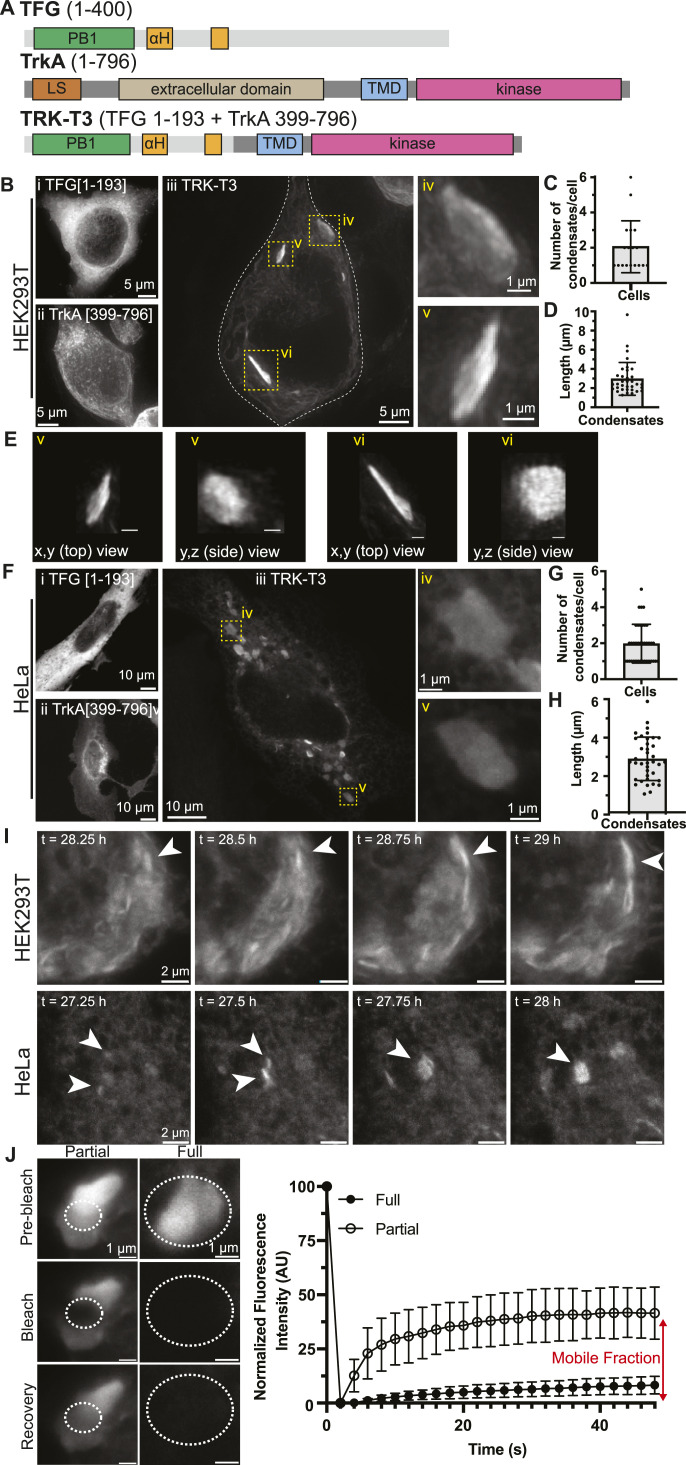
TRK-T3 forms biomolecular condensates. **(A)** Schematic depicting the domains of TFG, TrkA, and their oncogenic fusion TRK-T3. PB1 domain, green; alpha helices, orange; hydrophobic transmembrane domain, light blue; kinase domain, pink. **(B)** Representative widefield deconvolution micrographs of HEK293T cells expressing TFG (1–193)-mEGFP-FLAG (i), TrkA (399–796)-mEGFP-FLAG (ii), and TRK-T3-mEGFP-FLAG (iii). Scale bar: 5 μm. Insets of magnified TRK-T3-mEGFP-FLAG domains, iv and v. Scale bar: 1 μm. **(C)** Quantification of the number of condensates per cell of TRK-T3-mEGFP-FLAG, 2 ± 1, n = 18 cells. **(D)** Quantification of size, longest distance, of TRK-T3-mEGFP-FLAG condensates, 3.0 μm ± 1.7, n = 34 condensates. **(E)** Volumetric reconstruction of sheet-like TRK-T3-mEGFP-FLAG domains, v and vi. Shown in (x, y) and (y, z) planes as indicated. Scale bar: 1 μm. **(F)** Representative micrographs of HeLa cells expressing TFG (1–193)-mEGFP-FLAG (i), TrkA (399–796)-mEGFP-FLAG (ii), and TRK-T3-mEGFP-FLAG (iii). Scale bar: 10 μm. Insets of magnified TRK-T3-mEGFP-FLAG domains iv and v. Scale bar: 1 μm. **(G)** Quantification of the number of condensates per cell of TRK-T3-mEGFP-FLAG, 2 ± 1, n = 38 cells. **(H)** Quantification of size, longest distance, of TRK-T3-mEGFP-FLAG condensates, 2.8 μm ± 1.1, n = 38 condensates. **(I)** Time-lapse micrographs showing formation of TRK-T3-mEGFP-FLAG condensates in HEK293T cells (top panels) and HeLa cells (bottom panels). Arrowheads indicate condensate formation. Scale bar: 2 μm. **(J)** Left: representative confocal micrographs pre-bleach, bleached, and recovery used for FRAP for TRK-T3-mEGFP-FLAG condensates, partial and full. Scale bar: 1 μm. Right: quantification of average condensate recovery rates for partial FRAP (open circles), n = 26 condensates, full FRAP, n = 33 condensates (closed circles). Mobile fraction indicated with a red double-pointed arrow (partial ≈ 40%, full ≈ 8%). Circles represent means and error bars indicate SD. Source data are available for this figure.

We set out to test whether the oncogenic fusion protein TRK-T3 can form biomolecular condensates. TRK-T3 forms a 68 kD, 590 amino-acid protein from the first 193 N-terminal amino acids of TFG and the last 397 C-terminal amino acids of TrkA (Fig 1A) (Greco et al, 1995). The TFG portion of TRK-T3 contains a PB1 domain, predicted to facilitate homo-oligomerization (Witte et al, 2011), and two amphipathic alpha helices (Bogus et al, 2025). The TrkA portion of TRK-T3 contains the transmembrane domain (Franco et al, 2020) and the kinase domain; however, it does not contain a signal sequence, resulting in a cytosolic fusion protein (Greco et al, 1995). TFG has been shown to phase-separate into anisotropic hollow condensates whose mechanism of assembly is driven by hydrophobic sticker residues (Bogus et al, 2025). Given recent findings that oncogenic fusion proteins can phase-separate, we hypothesized that TRK-T3 would also be able to phase-separate and that this could be its mechanism of transformation (Tulpule et al, 2021; Zhu et al, 2023). To test for condensation of TRK-T3, we overexpressed recombinant TRK-T3 in both HEK293T and HeLa cells to explore putative differences between immortalized embryonic and cancer-derived cell lines, mimicking the approach used by other groups studying phase-separating oncogenic fusions (Zhu et al, 2023), as TRK-T3 cancer cell model lines are unavailable. TRK-T3-mEGFP-FLAG was transiently transfected and subjected to fluorescence microscopy after 24 h of expression (Fig 1Biii and Fiii). Strikingly, we observed that TRK-T3 assembled into sheet-like domains several micrometers in length (HEK: 3.0 μm ± 1.7, n = 34 condensates, Fig 1D; HeLa: 2.8 μm ± 1.1, n = 38 condensates, Fig 1E and H), as well as a faint localization pattern throughout the cell reminiscent of ER morphology and putatively indicative of a two-phase coexistence. TRK-T3 formed ∼2 prominent micron-sized domains per cell (HEK: 2 ± 2, n = 18 cells, Fig 1Biv and v and C; HeLa: 2 ± 1, n = 38 cells, Fig 1Fiv and v and G), suggesting the sequestration of recombinant proteins into those structures. Importantly, the morphology of TRK-T3 domains did not resemble protein networks formed by full-length TFG, which assembles into sub-micron-sized condensates that are anisotropic hollow spheres (Bogus et al, 2025). Although the morphology of TFG condensates was preserved across a variety of different TFG truncations (Bogus et al, 2025), we next tested whether the individual TFG or TrkA fragments present in TRK-T3 were able to produce the sheet-like domains observed for the full-length oncoprotein. Upon overexpression of the recombinant proteins corresponding to the component parts of TRK-T3, TFG(1–193), and TrkA(399–796), we found that neither moiety on its own forms domains resembling those formed by full-length TRK-T3. The TFG(1–193) moiety appeared dispersed throughout the cytosol, lacking both the domain and ER-like faint localization pattern observed for TRK-T3 (Figs 1Bi and Fi and S1A and B, top panels), whereas the TrkA(399–796) moiety formed amorphous foci/aggregates that appeared enriched in the perinuclear region (Fig 1Bii and Fii and S1A and B, bottom panels). These data suggest that both moieties of the TRK-T3 fusion protein contribute to the formation of sheet-like domains.

**Figure S1.**
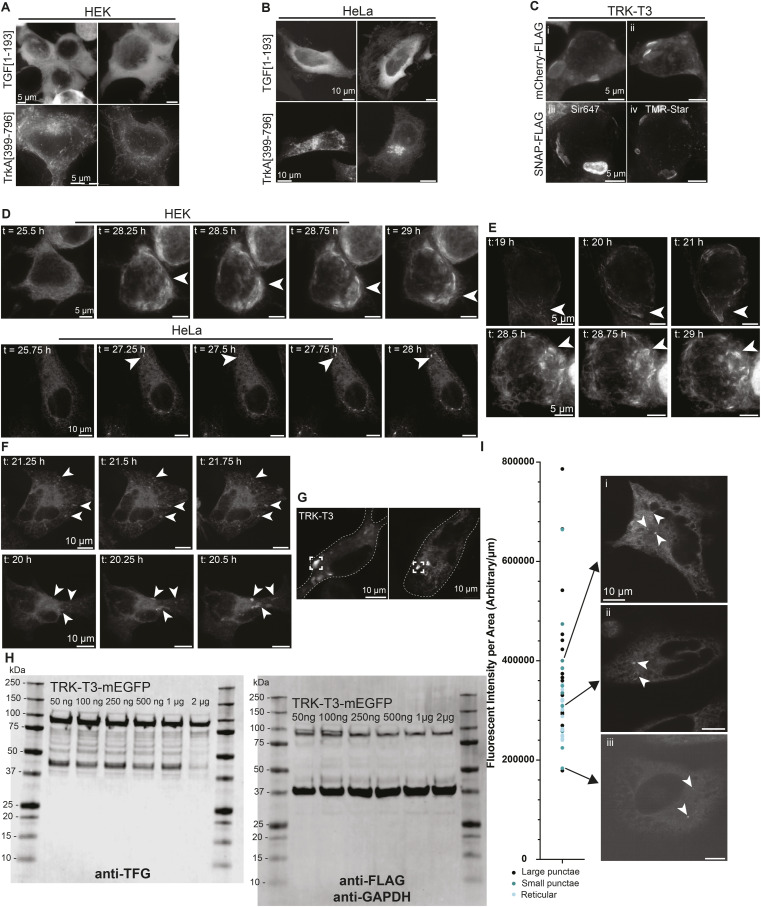
Condensate formation of TRK-T3 over time and expression compared with endogenous TFG. **(A)** Representative widefield deconvolution micrographs of HEK293T cells expressing TFG(1–193)-mEGFP-FLAG (top panels) and TrkA(399–796)-mEGFP-FLAG (bottom panels). Scale bar: 5 μm. **(B)** Representative confocal micrographs of HeLa cells expressing TFG(1–193)-mEGFP-FLAG (top panels) and TrkA(399–796)-mEGFP-FLAG (bottom panels). Scale bar: 10 μm. **(C)** Representative micrographs of HEK293T cells expressing TRK-T3-mCherry-FLAG (top panels, confocal microscopy) and TRK-T3-SNAP-FLAG (widefield deconvolution microscopy), stained with SNAP-SiR647 (iii) or SNAP-TMR-Star (v) (bottom panels). Scale bar: 5 μm. **(D)** Overviews of HEK293T cell (top panels) and HeLa cell (bottom panel) used in Fig 1H. Arrowheads indicate condensates forming. Scale bar in the top panels (HEK293T): 5 μm. Scale bar in the top panels (HeLa): 10 μm. **(E)** Representative confocal micrographs of two individual HEK cells expressing TRK-T3-mEGFP-FLAG over time. Arrowheads indicate condensates forming. Scale bar: 5 μm. **(F)** Representative confocal micrographs of two individual HeLa cells expressing TRK-T3-mEGFP-FLAG over time. Arrowheads indicate condensates forming. Scale bar: 10 μm. **(G)** Cell overviews of HeLa cells used in FRAP of Fig 1I. Scale bar: 10 μm. **(H)** Representative Western blots of HeLa TFG::mClover-FLAG (heterozygous) expressing increasing levels of TRK-T3-mEGFP-FLAG. Top blot: anti-TFG, bottom blot: anti-GAPDH and anti-FLAG. **(I)** Left: graph of integrated density over area of cell. Black dots represent cells with large domains and reticular patterning, dark teal dots represent cells with small domains and reticular patterning, cyan dots represent cells with only reticular phenotype. Representative confocal micrographs of HeLa cells expressing TRK-T3 at increasing intensities from bottom to top. Arrows indicate which datapoint micrographs originate from, and arrowheads indicate small domains. Scale bar: 10 μm. Source data are available for this figure.

Next, we measured the expression level of transiently transfected TRK-T3-mEGFP-FLAG and compared it to an endogenously tagged TFG::mClover-FLAG (Fig S1H). Endogenous TFG is able to form condensates, with saturation concentrations found to be an order of magnitude below cellular levels in vitro (Bogus et al, 2025). We find that at the highest expression level of 2 μg of TRK-T3, there is approximately an eightfold increase in expression when compared with endogenous tagged TFG expression. As performed in previous studies using overexpression of TRK-T3 (Greco et al, 1995; Witte et al, 2011; Zhu et al, 2023), for our characterization, this enables characterization of TRK-T3 assemblies via fluorescence microscopy. Furthermore, we find that HeLa cells expressing low amounts of TRK-T3, as measured by total cell integrated intensity divided by cell area, support the formation of dynamic assemblies that are somewhat smaller than those observed with higher expression (Fig S1I).

To examine how the sheet-likeTRK-T3 domains appeared in cells, we performed time-lapse confocal fluorescence microscopy of HEK293T and HeLa cells transfected with TRK-T3-mEGFP-FLAG over 9–12 h, acquiring Z-stacks in 15-minute intervals. TRK-T3 initially exhibited a faint ER-like, reticular staining pattern (Fig S1D, top: HEK293T t = 25.5 h, bottom: HeLa t = 25.75 h). Over the course of 1-h sheet-like domains abruptly appeared, which was not restricted to the cell type (Figs 1I and S1D, HEK293T t = 28.25–29 h, HeLa t = 27.25–28 h, Fig S1E, HEK293T, and Fig S1F, HeLa), and might represent a phase transition, in which the dilute TRK-T3 phase unmixes into a two-phase coexistence (Banani et al, 2017).

Because a recent publication highlighted the impact of protein tags on protein condensation (Dörner et al, 2024 *Preprint*), we generated additional fusions of TRK-T3. HEK293T cells were transfected with TRK-T3-mCherry-FLAG or TRK-T3-SNAP-FLAG (labeled with SiR647 or TMR-Star) for 24 h and subjected to confocal/widefield microscopy (Fig S1C). Importantly, both alternative fusion proteins formed sheet-like domains, at a similar frequency per cell and with similar dimensions (mCherry fusion: number of condensates per cell = 2 ± 1, n = 8 cells, condensate length = 3.9 μm ± 1.3, n = 16 condensates; SNAP fusion: number of condensates per cell = 1 ± 1, n = 5 cells, condensate length = 5.64 μm ± 2.03, n = 8 condensates), compared with TRK-T3 fused to mEGFP.

To probe whether the planar domains formed by TRK-T3 might represent biomolecular condensates, we set out to test their fluidity by using FRAP (Fig 1J; cell overview Fig S1G). We transfected HeLa cells with TRK-T3-mEGFP-FLAG and performed partial and full FRAP of sheet-like domains at 24-h post-transfection. In partial FRAP experiments, TRK-T3 domains recovered maximally within seconds, suggesting a great extent of diffusion within the condensate dense phase (∼40%; Fig 1J, right graph; mobile phase, indicated by double-pointed red arrow). The recovery of ∼40% of fluorescence is consistent with a largely liquid-like condensate, considering that ∼50% of the condensate area was completely bleached. In photobleaching entire condensates (full FRAP), TRK-T3 domains did not significantly recover on the same time scale, suggesting low molecule mobility between the dense and dilute phases, and suggesting that the recovery during the partial bleaching is because of internal fluidity of the condensate, rather than molecule turnover by exchange with the surrounding medium.

### TRK-T3 condensates accumulate in the vicinity of the PM

**Figure 2.**
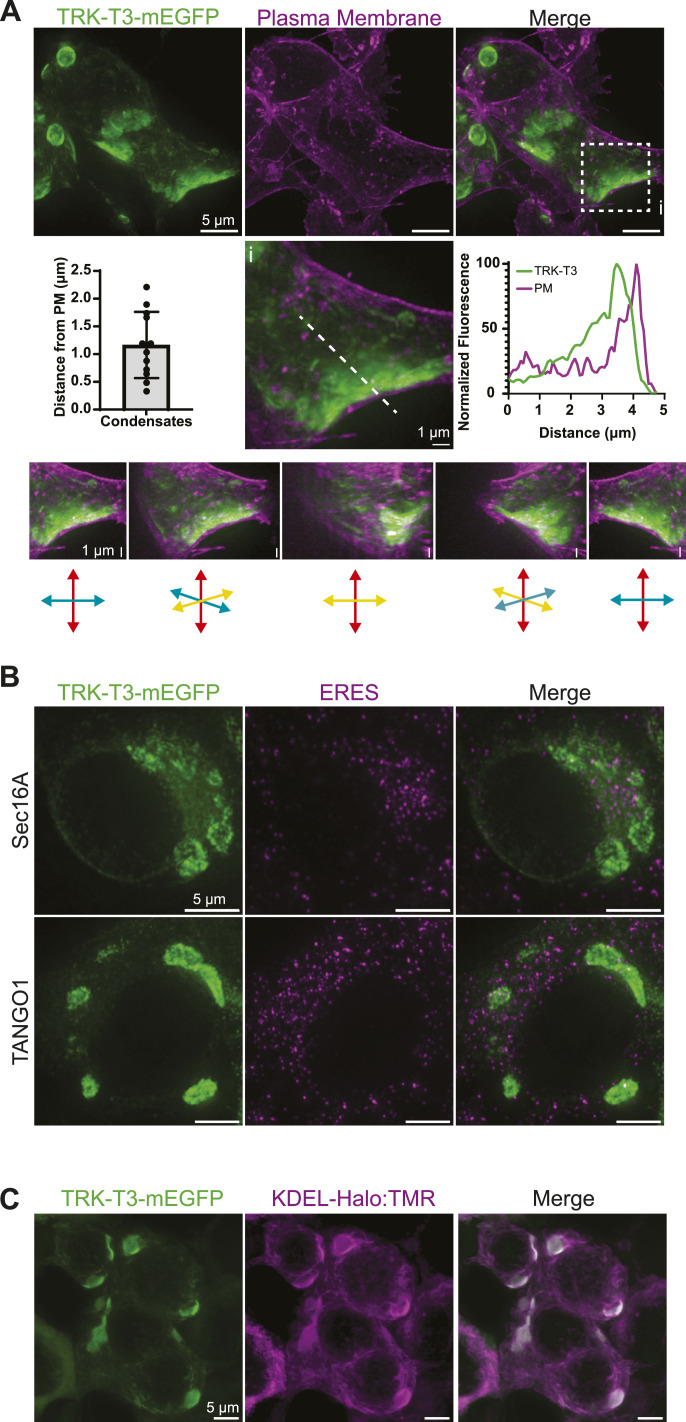
TRK-T3 localizes to the plasma membrane. **(A)** Representative widefield deconvolution micrograph of HEK293T cells transfected with 1 μg of TRK-T3-mEGFP-FLAG (green) for 24 h and 30 particles per cell CellLight Plasma Membrane Red (magenta) for 18 h. Middle: quantification of distance between edge of sheet-like condensate and plasma membrane (left) (n = 11 condensates); magnification of condensate (middle); fluorescence intensity line scan of middle panel (right). Scale bar: 5 μm. Bottom: volumetric reconstruction of the magnification of condensate from above, presented at various angles. Orientations are indicated as x (blue), y (red), and z (yellow). Scale bar: 1 μm. **(B)** Representative widefield deconvolution micrographs of HEK392T cells transfected with 1 μg of TRK-T3-mEGFP-FLAG for 24 h and immunostained with ER exit site markers (ERES) Sec16A (top) and Tango1 (bottom). Scale bar: 5 μm. **(C)** Representative confocal micrographs of HEK293T cells co-transfected with 1 μg of TRK-T3-mEGFP and 1 μg of KDEL-Halo stained with 1 μM of Halo-TMR ligand and expressed for 24 h. Scale bar: 5 μm. Source data are available for this figure.

**Figure S2.**
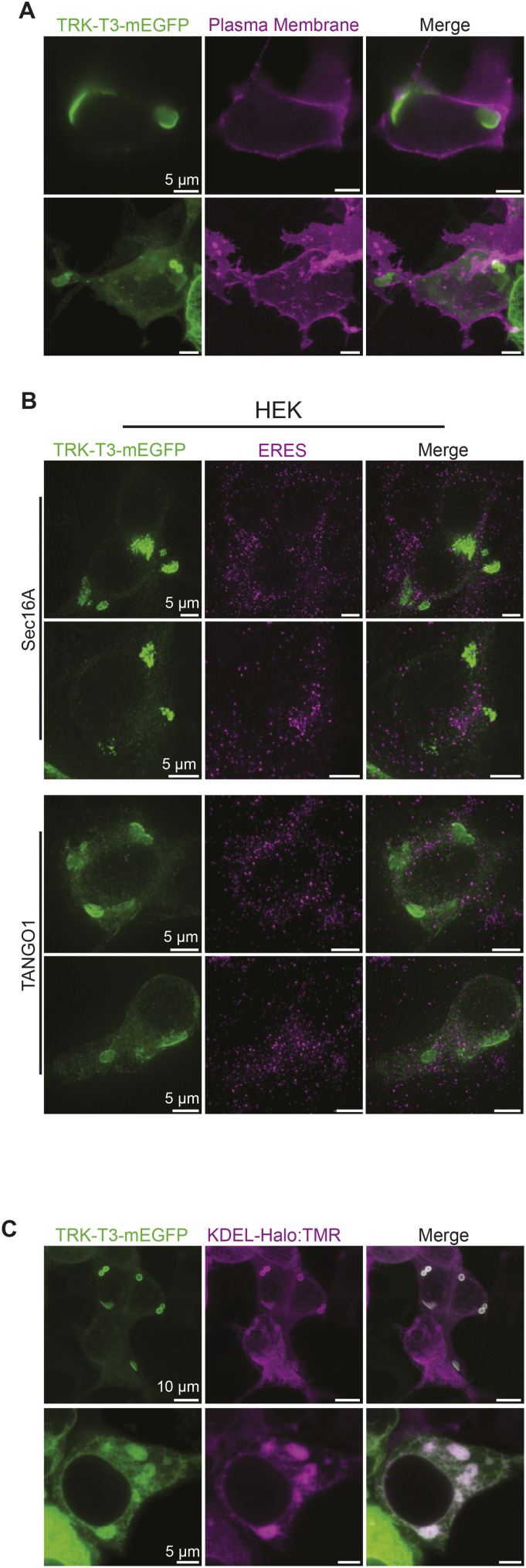
TRK-T3 localizes to the plasma membrane and ER but not ER exit sites. **(A)** Representative widefield micrographs of HEK293T cells transfected with 1 μg of TRK-T3-mEGFP-FLAG (green) for 24 h and 30 PPC CellLight Plasma Membrane Red (magenta) for 18 h. Scale bar: 5 μm. **(B)** Representative widefield deconvolution micrographs of HEK392T cells transfected with 1 μg of TRK-T3-mEGFP-FLAG for 24 h and immunostained with ERES markers Sec16A (top rows) and TANGO1 (bottom rows). Scale bar: 5 μm. **(C)** Representative confocal micrographs of HEK293T cells expressing 1 μg of TRK-T3-mEGFP-FLAG and 1 μg KDEL-Halo stained with 1 μM of Halo-TMR ligand at 24 h. Scale bar top: 10 μm, bottom: 5 μm.

We observed that TRK-T3 condensates appeared enriched towards the cell periphery, suggesting their accumulation at the PM. Localization to the PM could allow TRK-T3 condensates to recruit key signal-transduction molecules, such as Ras proteins that are peripherally anchored to the cytosolic leaflet of the PM, thus potentially contributing to TRK-T3-induced cell transformation and oncogenesis. To assess its subcellular localization, we transfected HEK293T cells with TRK-T3-mEGFP-FLAG, and after 8 h, used the CellLight system at a concentration of 30 particles per cell (PPC) to stain the PM (Figs 2A and S2A). At 24-h post-transfection, we found that although the TRK-T3 sheet-like condensates are indeed enriched near the PM, they did not colocalize fully, rather exhibiting an average distance of 1 μm from the PM (Fig 2A, middle panels, 1.2 μm ± 0.6, n = 11 condensates), which may suggest exclusion by cytoskeletal networks, or putatively an active transport towards the PM, for example, via kinesin motors on microtubules. Volumetric reconstructions elucidate proximity at various angles (Fig 2A bottom panels).

Localization of TRK-T3 condensates to the PM implies two important steps in the initiation of TRK-T3-induced cell transformation. First, condensation of TRK-T3 into sheet-like condensates could bring the kinase domains encoded in the TrkA moiety into close proximity, triggering an extracellular ligand-independent autophosphorylation of kinase domains, which was reported recently (Toledo et al, 2023). Second, the condensate dense phase could promote a sequestration of signal-transduction molecules, beyond a stoichiometric recruitment to kinase domains that are in the vicinity of the PM into the condensate dense phase, thus leading to an efficient extracellular ligand-independent activation of growth signaling and cell transformation (Cocco et al, 2018).

Previous subcellular localization efforts identified TRK-T3 at ER exit sites (ERES), prompting a model in which a feedforward mechanism results in activation of the MAPK pathway and oncogenesis (Witte et al, 2011). In that work, overexpressed recombinant Sec16A was used as a marker for ERES, resulting in a high colocalization with recombinant TRK-T3 upon co-overexpression. Because Sec16A itself forms condensates (Gallo et al, 2023), and TFG interacts with Sec16 (Witte et al, 2011), we set out to test whether TRK-T3 would exhibit colocalization with endogenous ERES markers. We transfected HEK293T cells with TRK-T3-mEGFP-FLAG for 24 h, followed by immunolabeling with either anti-Sec16A or anti-Tango1 antibodies after fixation, fluorescence microscopy, and colocalization analysis. We could not detect any significant colocalization with endogenous ERES markers Sec16 or TANGO1 (Sec16A: Figs 2B and S2B top panels, Pearson’s coefficient = 0.055 ± 0.302, n = 7 cells; TANGO1: Figs 2B and S2B bottom panels, Pearson’s coefficient = 0.393 ± 0.148; n = 9 cells). In addition, when we co-transfect HEK293T cells with TRK-T3-mEGFP and KDEL-Halo, a marker for the ER, we find that TRK-T3 colocalizes well with the KDEL signal (Fig 2C, Pearson’s coefficient = 0.889 ± 0.084, n = 14 cells; Fig S2C). Together, these data point towards accumulation of TRK-T3 at the ER, but not at ERES exclusively. Accumulation of TRK-T3 at the ER could be contributing to condensation by locally increasing concentrations of TRK-T3 and thus resulting in its phase transition. It remains unclear how TRK-T3 condensates then accumulate in the vicinity of the PM.

### TRK-T3 condensate morphology is mediated by a hydrophobic moiety

**Figure 3.**
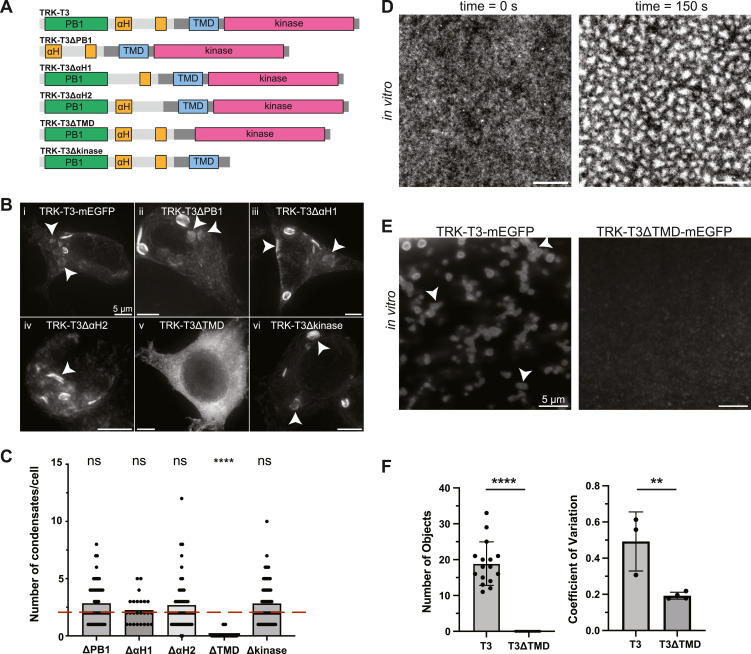
TRK-T3 condensate morphology depends on a hydrophobic segment. **(A)** Schematic depicting domain deletions of TRK-T3. PB1 domain (AA 10–91), green; alpha helices (ɑH1—AA 99–123; ɑH2—AA 174–184), orange; hydrophobic transmembrane domain (AA 209–234), light blue; kinase domain (AA 305–575), pink. **(B)** Representative widefield deconvolution micrographs of HEK293T cells transfected with domain deletions of TRK-T3-mEGFP-FLAG at 1 μg for 24 h. Scale bar: 5 μm. **(C)** Quantification of condensates/cell for each domain deletion variant, TRK-T3: 2 ± 1 (dotted line, Fig 1C, n = 18 cells), ΔPB1: 3 ± 2, n = 55 cells, *P* = 0.0717, ΔɑH1: 2 ± 1, n = 25 cells, *P* = 0.6652, ΔɑH2: 3 ± 2, n = 53 cells, *P* = 0.1832, ΔTMD: 0 ± 0, n = 38 cells, *P* = <0.0001, Δkinase: 3 ± 2, n = 72 cells, *P* = 0.0696. *P*-values determined by individual Welch’s two-tailed *t* tests comparing domain deletion variant to full-length TRK-T3 number of condensates/cell. **(D)** Representative TIRF micrographs of purified recombinant TRK-T3-mEGFP-FLAG exhibiting phase transition during sessile droplet evaporation. Scale bar: 5 μm. **(E)** Representative confocal micrographs of purified recombinant TRK-T3-mEGFP-FLAG (left) and TRK-T3ΔTMD-mEGFP-FLAG (right). Arrowheads point to putative fusion events. Scale bar: 5 μm. See the Materials and Methods section. **(F)** Quantification of the number of condensates (left) and coefficient of variation, which is a ratio of SD and average fluorescence intensity of a representative area of a micrograph (right) of TRK-T3-mEGFP-FLAG and TRK-T3ΔTMD-mEGFP-FLAG in vitro. Number of objects: T3, n = 16; T3ΔTMD, n = 19, *P* < 0.0001. Coefficient of variation: regions of interest of the same size; T3, n = 3; T3ΔTMD, n = 4, *P* = 0.0052. *P*-values determined by Welch’s two-tailed *t* test. Source data are available for this figure.

**Figure S3.**
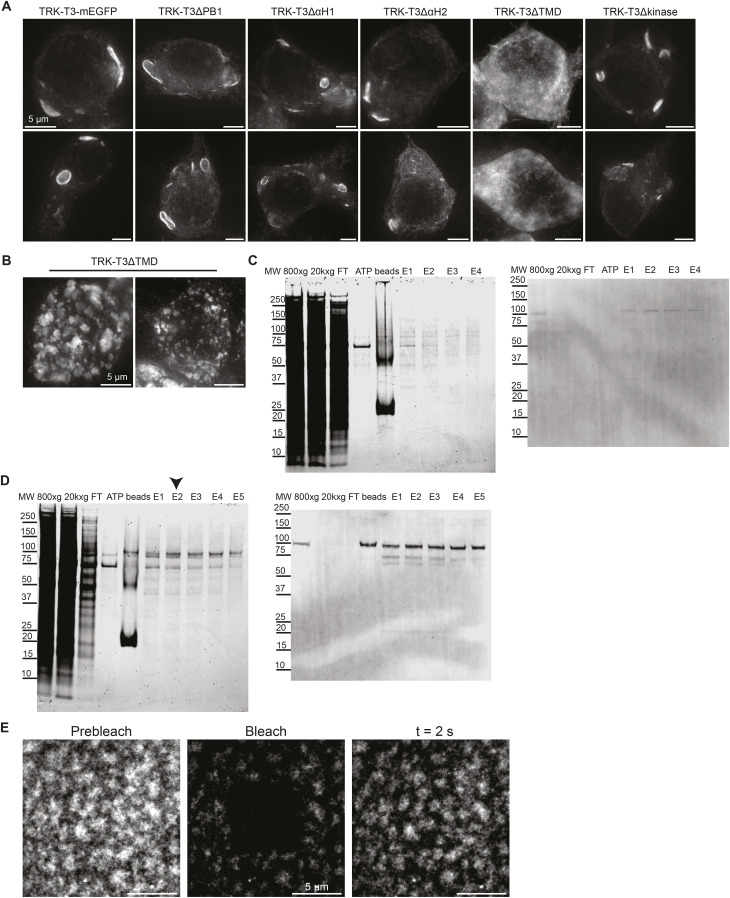
TRK-T3 condensation depends on a hydrophobic domain in cells and in vitro. **(A)** Representative widefield deconvolution micrographs of TRK-T3-mEGFP deletion variants in HEK293T cells. Scale bar: 5 μm. **(B)** Representative widefield deconvolution micrographs of TRK-T3ΔTMD-mEGFP in HEK293T cells that form condensate-like domains. Scale bar: 5 μm. **(C)** Representative purification overview of TRK-T3-mEGFP-FLAG with Coomassie stain (left) and corresponding anti-GFP Western blot of TRK-T3-mEGFP-FLAG (right). All elutions pooled for use. **(D)** Representative purification overview of TRK-T3ΔTMD-mEGFP-FLAG with Coomassie stain (left) and corresponding anti-GFP Western blot of TRK-T3ΔTMD-mEGFP-FLAG (right). The arrow indicates the elution used. **(E)** Qualitative FRAP of TRK-T3-mEGFP-FLAG condensates in vitro. Scale bar: 5 μm.

Next, we set out to test whether specific domains within the fusion protein are responsible for the formation of TRK-T3 condensates by individually deleting the five folded domains of TRK-T3: the Phox and Bem 1 (PB1), alpha helix 1 (aH1), alpha helix 2 (aH2), transmembrane (TMD), and kinase (kin) domains (Fig 3A). We transiently transfected the respective recombinant TRK-T3 domain deletion variants into HEK293T cells and subjected them to fluorescence microscopy 24-h post transfection. We find that deleting the PB1, alpha helices, or kinase domains did not qualitatively abolish condensate formation by TRK-T3 variants (Figs 3Bii, iii, iv, and vi and S3A). We also could not detect significant differences in the number of condensates per cell in the ΔPB1, ΔαH1, ΔαH2, and Δkinase deletions, compared with full-length TRK-T3 (Fig 3C; ΔPB1 condensates/cell = 3 ± 2, *P* = 0.0717, n = 55 cells, ΔαH1 condensates/cell = 2 ± 1, *P* = 0.6652, n = 25 cells, ΔαH2 condensates/cell = 3 ± 2, *P* = 0.1832, n = 53 cells, Δkinase condensates/cell = 3 ± 2, *P* = 0.0696, n = 72 cells). Strikingly, deleting the transmembrane domain encoded in the TrkA moiety largely abolished the formation of TRK-T3 condensates (Figs 3Bv and S3A; Fig 3C, ΔTMD condensates/cell = 0 ± 0, *P* = <0.0001, n = 38 cells). Instead, cells expressing TRK-T3ΔTMD exhibited a hazy, non-reticular cytosolic distribution, with only a small fraction of transfected cells exhibiting foci that did not resemble sheet-like TRK-T3 condensates (Fig S3B). This suggests that the TMD within the TrkA moiety of the oncoprotein strongly impacts TRK-T3 condensate morphology, resulting in sheet-like condensates unlike the hollow spheres formed by TFG (Bogus et al, 2025).

Next, we examined the intrinsic ability of TRK-T3 to form condensates. Although expressing recombinant TRK-T3 within cells enabled screening of the propensity of the oncoprotein to associate into condensates within a crowded cellular environment, it cannot be excluded that unknown cytosolic factors may be required or contribute to TRK-T3 condensation. We obtained recombinant TRK-T3-mEGFP-FLAG from Expi293f human suspension cells (Fig S3C) and screened for its ability to undergo a LLPS using a sessile droplet evaporation assay (Rebane et al, 2020) in which the target protein is probed along a concentration gradient for its ability to form condensates, and in the absence of crowding agents or other cofactors (see the Materials and Methods section). Importantly, upon using time-lapse microscopy, we observe abrupt phase transitions of TRK-T3 into dynamic condensates, before supersaturation, with the resulting condensates exhibiting hallmarks of fluidity, such as recovery upon photobleaching (Figs 3D and S3E). Strikingly, full-length TRK-T3 readily associated to form rounded, flattened condensates that strongly reflected the condensate morphology observed in cells (in vitro: Fig 3E, left; in cells: Fig 1Biii and Eiii). Importantly, TRK-T3 condensates appeared to be able to fuse together to form irregularly shaped structures, highlighting the putative viscoelastic properties of the condensate dense phase (Fig 1E, arrowheads). To test whether the TMD encoded in the TrkA moiety of the oncoprotein impacted phase separation of TRK-T3 in vitro, we purified TRK-T3ΔTMD-mEGFP-FLAG (Fig S3D). In the absence of the TMD, the protein was unable to form condensates (Fig 3D, right, Fig 3E), instead dispersing nonspecifically on the glass surface, which strongly parallels the haze formed by the TMD-lacking TRK-T3 variant overexpressed in cells. Disassembly of condensates in the absence of the TMD was further reflected by the significantly reduced coefficient of variation (Fig 3E), which is calculated as the ratio between the SD and the average fluorescence intensity across the micrographs, with greater values indicating greater degrees of condensation as reported previously (Bracha et al, 2018). We found that TRK-T3 condensates formed in vitro were significantly smaller than those formed in cells (in vitro condensate size = 0.6 μm ± 0.2, [n = 95 condensates, Fig 3E], condensate size in cells = 3.0 μm ± 1.7, n = 34 condensates [Fig 1D and G]), suggesting that stabilization of sheet-like condensates in proximity of the PM because of cytoskeletal elements or interaction with signaling molecules could further impact condensate dimensions.

### Recruitment of signal-transduction molecules to TRK-T3 condensates requires a sheet-like morphology

**Figure 4.**
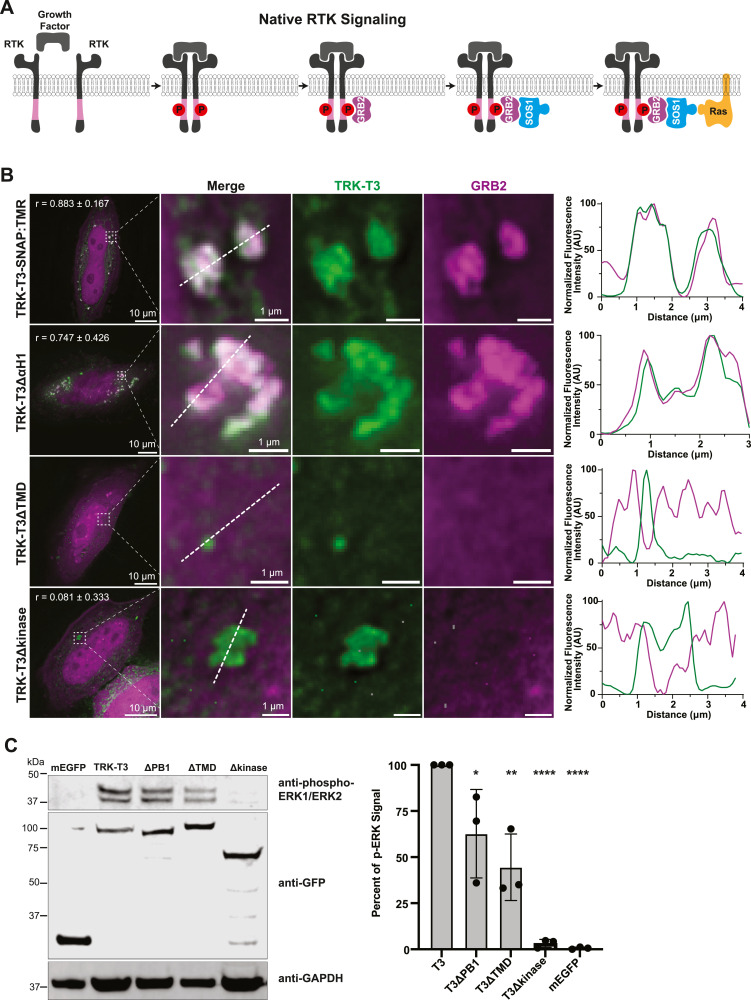
TRK-T3 signal-transduction molecule recruitment and MAPK activation is dependent on condensate morphology. **(A)** Schematic depicting native RTK signaling. **(B)** Representative maximum intensity Z-projections of widefield deconvolution micrographs of TRK-T3-SNAP-FLAG domain deletion variants co-expressed with GRB2-EGFP in HeLa cells, TRK-T3 variants transfected at 2 μg, and GRB2 transfected at 0.5 μg. Cells were stained with SNAP-TMR-Star ligand at 1 μM. Scale bar: 5 μm. r = Pearson correlation coefficient for condensates; TRK-T3, n = 30 condensates, TRK-T3ΔαH1, n = 66 condensates, TRK-T3Δkinase, n = 35 condensates. **(C)** Representative Western blots of HEK293T cells transiently overexpressed with mEGFP-FLAG, TRK-T3-mEGFP-FLAG, TRK-T3-ΔPB1-mEGFP-FLAG, TRK-T3-ΔTMD-mEGFP-FLAG, and TRK-T3Δkinase-mEGFP-FLAG. Samples are load-adjusted to similar GFP expression levels. Top blot: anti-phospho-ERK, middle blot: anti-GFP, bottom blot: anti-GAPDH. Right: quantification of intensity of bands, normalized to TRK-T3, expressed as percent of p-ERK signal, n = 3 blots, ΔPB1: *P* = 0.0221, ΔTMD: *P* = 0.0017, Δkinase: *P* < 0.0001, mEGFP: *P* < 0.0001. *P*-values calculated by Welch’s two-tailed *t* test. Source data are available for this figure.

**Figure S4.**
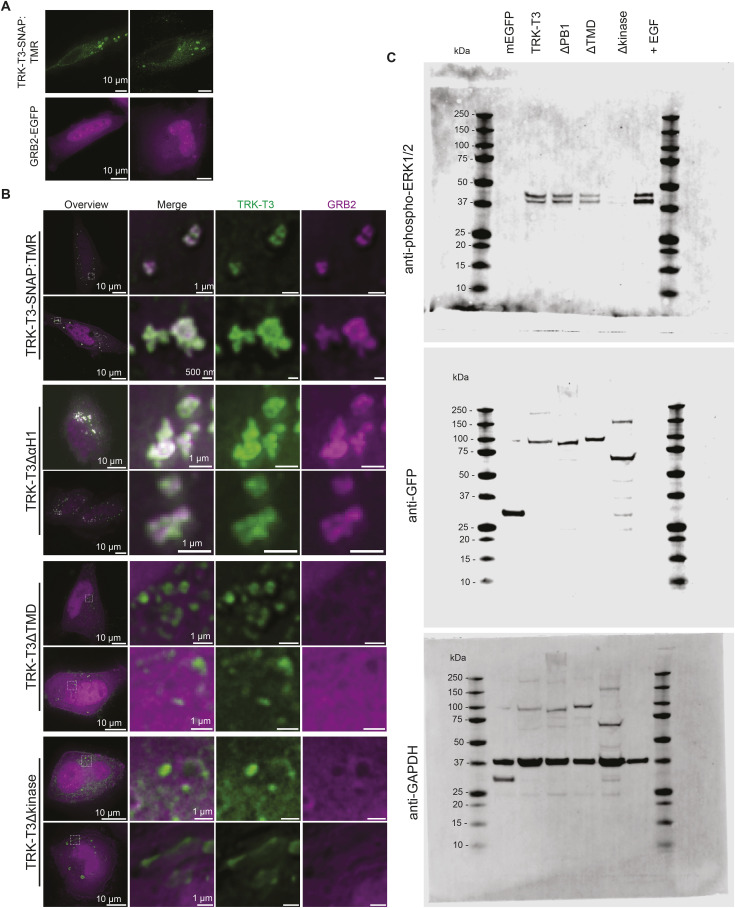
TRK-T3 condensates GRB2 recruitment depends on condensate morphology. **(A)** Representative widefield deconvolution micrographs of HeLa cells transfected with 2 μg of TRK-T3-SNAP-FLAG, labeled with 1 μM of TMR-Star ligand (top) and 0.5 μg of GRB2-EGFP (bottom). **(B)** Representative widefield deconvolution micrographs of HeLa cells co-transfected with 2 μg of TRK-T3-SNAP-FLAG and 0.5 μg of GRB2-EGFP. Cells stained with 1 μM of TMR-Star SNAP ligand. Scale bar: 10 μm. **(C)** Representative Western blots for anti-p-ERK (top), anti-GFP (middle), and anti-GAPDH (bottom) from Fig 4C.

The proximity of kinase domains within the TRK-T3 condensate dense phase was expected to favor tyrosine autophosphorylation and, thus, putatively result in the recruitment of signal-transduction molecules. Previous studies have reported that other oncogenic RTK domain-containing fusion proteins are able to sequester signal-transduction molecules such as GRB2 and SOS1 (Tulpule et al, 2021; Gao et al, 2025 *Preprint*). The GRB2 SH2 domains bind to phosphorylated tyrosine residues on kinase domains and act as an adaptor protein between phosphorylated RTK proteins and SOS1, a guanine nucleotide exchange factor. SOS1 then interacts with Ras to activate signaling pathways (Guo et al, 2020). We hypothesized that the putatively planar condensate geometry of TRK-T3 could allow for an efficient autophosphorylation because of their alignment, analogously to the autophosphorylation that occurs when native RTKs dimerize within the PM upon ligand binding (Fig 4A). To examine if TRK-T3 can recruit GRB2, we co-transfected TRK-T3-SNAP-FLAG and GRB2-EGFP into HeLa cells. To visualize the TRK-T3 recombinant protein, we labeled the cells with the cell-permeable SNAP ligand TMR-Star 24-h post-transfection and then subjected the cells to fluorescence microscopy. Although their morphology was retained, we found that in the presence of GRB2, TRK-T3 condensates were smaller in cells (Fig 4B, top panels, Fig S4B, top two rows, condensate length = 1.8 μm ± 0.7, n = 33 condensates). TRK-T3, in the absence of overexpressed GRB2-EGFP, formed condensates comparable in morphology and dimension compared with mEGFP fusions (Fig S4A, top, condensate length = 3.3 μm ± 0.9, n = 30 condensates). Strikingly, TRK-T3 condensates in HeLa cells strongly colocalized with GRB2, suggesting efficient autophosphorylation of TRK-T3 and recruitment of GRB2 to the condensate dense phase. We measured this by quantifying the Pearson’s correlation coefficient of TRK-T3 condensates with GRB2 signal (Figs 4B and S4B; Pearson’s coefficient = 0.883 ± 0.167, n = 98 condensates). Next, we tested whether the condensate morphology favored recruitment of GRB2 and whether its recruitment depended on the kinase domains within TRK-T3 (rather than nonspecific recruitment to the dense phase) by co-transfecting GRB2 with variants TRK-T3ΔαH1, TRK-T3ΔTMD, and TRK-T3Δkinase. We find that cells expressing TRK-T3ΔαH1 retain high colocalization with GRB2 (Figs 4B and S4B; Pearson’s coefficient = 0.747 ± 0.426, n = 66 condensates). Furthermore, we find that although TRK-T3Δkinase is still able to form condensates, it fails to significantly recruit GRB2 (Figs 4B and S4B; Pearson’s coefficient = 0.081 ± 0.333, n = 35 condensates). Because TRK-T3ΔTMD does not form condensates, we instead measured the colocalization of TRK-T3ΔTMD with GRB2, which was low (Figs 4B and S4B; Pearson’s coefficient = 0.339 ± 0.153, n = 10 cells). These data highlight that the TMD favors condensate formation that can lead to an efficient autophosphorylation of kinase domains within the dense phase, leading to an efficient and specific recruitment of the first-layer signal-transduction molecule GRB2.

Previous literature indicates that TRK-T3 can activate the MAPK pathway, resulting in cell transformation (Greco et al, 1995; Witte et al, 2011). Beyond the recruitment of initial signal-transduction molecules to the TRK-T3 condensates, we set out to test their ability to activate the MAPK pathway by performing quantitative Western blots of HEK293T cell extracts overexpressing TRK-T3 or its domain deletion variants immunostaining for endogenous phosphorylated ERK (p-ERK) proteins, as p-ERK 1 and 2 are commonly used as a readout for the activation of the MAPK pathway (Witte et al, 2011). To control for the expression level of individual TRK-T3 and TRK-T3 variants, we immunostained for GFP. We quantified the extent of MAPK activation for full-length TRK-T3, TRK-T3ΔPB1, TRK-T3ΔTMD, and TRK-T3Δkinase, as well as mEGFP, as a negative control, and EGF-treated cells as a positive control. When normalized to the expression level of TRK-T3, we find reductions in p-ERK signal for TRK-T3 domain deletion variants. TRK-T3ΔPB1 is reduced by 37%, TRK-T3ΔTMD is reduced by 56%, and TRK-T3Δkinase is reduced by 97% (Fig 4C, right panel, n = 3 blots, Fig S4C). These data suggest that TRK-T3 condensation is directly linked to the extent of MAPK pathway activation, with the TFG PB1 domain putatively contributing to kinase domain alignment within condensates, and the TrkA greatly impacting the extent of autophosphorylation of kinase domains in the condensate dense phase. The residual ability of TRK-T3ΔTMD to activate the MAPK pathway may be because of its 10-fold higher expression in cells, favoring stochastic encounters of kinase domains in the cytosol and aggregates and, thus, resulting in a significant but less efficient autophosphorylation of kinase domains and activation of the MAPK pathway. Interestingly, we find that although the TRK-T3 variant lacking the PB1 domain is still able to adopt a sheet-like condensate morphology, it exhibits a significant reduction in ERK phosphorylation, which may point to a contribution of PB1 within the TFG moiety of the fusion protein and the alignment of molecules within the TRK-T3 condensate dense phase to enhance autophosphorylation and growth signaling.

### A minimal hydrophobic segment mimicking the TrkA transmembrane domain dissolves TRK-T3 condensates

**Figure 5.**
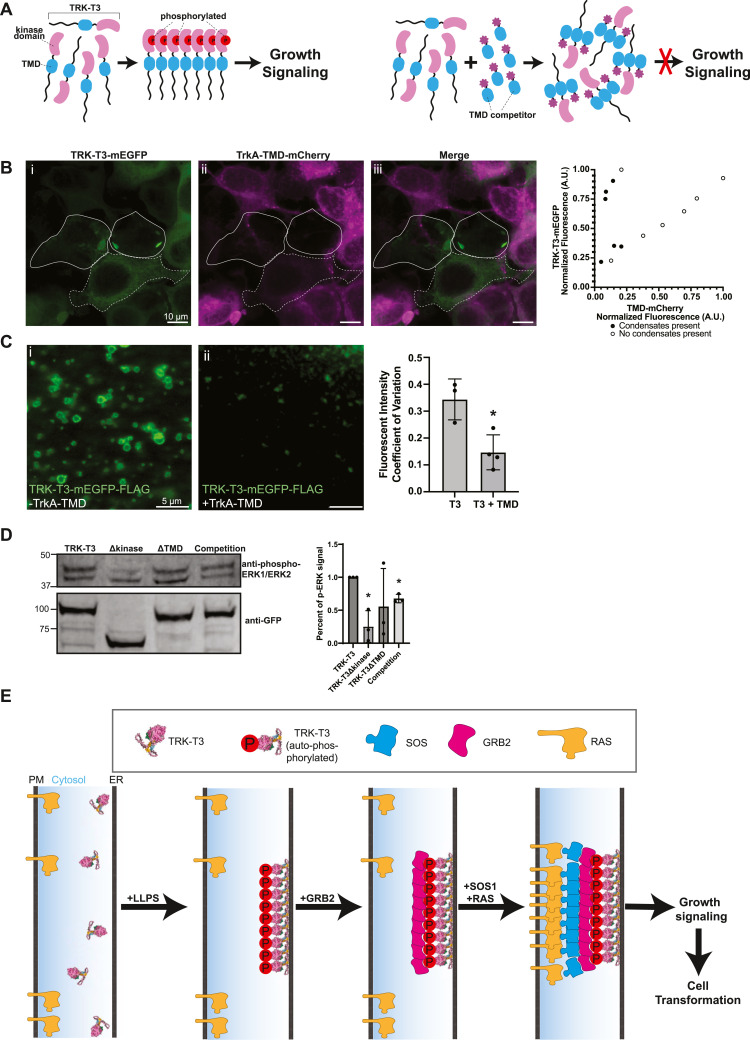
TRK-T3 condensates are disrupted by a hydrophobic segment mimicking the TrkA-TMD. **(A)** Schematic depicting TRK-T3 sheet-like condensate formation via TMD (left) versus TRK-T3 condensate formation being inhibited by TrkA-TMD-mCherry addition. **(B)** Representative widefield deconvolution micrograph of HEK293T cells co-expressing TRK-T3-mEGFP-FLAG and TrkA-TMD-mCherry-FLAG. TRK-T3-mEGFP-FLAG was transfected at 1 μg, and TrkA-TMD-mCherry-FLAG was transfected at 1 μg for 24 h. Scale bar: 10 μm. Plot depicting normalized TRK-T3-mEGFP fluorescent intensity versus normalized TMD-mCherry fluorescent intensity. Each dot represents one cell. Filled circles represent cells with condensates and unfilled circles represent cells without condensates. Cells with condensates outlined in solid white lines, cells with only reticular pattern outlined in dotted white lines. **(C)** Representative confocal micrographs of recombinant TRK-T3-mEGFP-FLAG and TrkA-TMD-mCherry-FLAG (see the Materials and Methods section). (i) 100% TRK-T3-mEGFP-FLAG. (ii) Green channel 50% TRK-T3-mEGFP-FLAG and 50% TrkA-TMD-mCherry-FLAG. Scale bar: 5 μm. Graph of fluorescent Intensity Coefficient of Variation, which is a ratio of SD and average fluorescent intensity of a representative area of a micrograph. Regions of interest of the same size; T3, n = 3; T3ΔTMD, n = 4, *P* = 0.0228. *P*-values calculated by Welch’s two-tailed *t* test. **(D)** Representative Western blots of HEK293T cells transiently overexpressed with TRK-T3-mEGFP-FLAG (24 h), TRK-T3Δkinase-mEGFP-FLAG (24 h), TRK-T3ΔTMD-mEGFP-FLAG (24 h), and TRK-T3-mEGFP-FLAG (24 h) with TrkA-TMD-mCherry (18 h). Samples are load-adjusted to similar levels of TRK-T3 and domain deletion variant expression. Top blot: anti-phospho-ERK, bottom blot: anti-GFP. Right: quantification of intensity of bands, normalized to TRK-T3, expressed as percent of p-ERK signal, n = 3 blots, Δkinase: *P* = 0.0329, ΔTMD: *P* = 0.3142, competition: *P* = 0.0130. *P*-values calculated by Welch’s two-tailed *t* test. **(E)** Model of TRK-T3 activation via GRB2 recruitment leading to propagation of signal transduction, including potential membrane wetting. TRK-T3 model generated by AlphaFold3. Source data are available for this figure.

**Figure S5.**
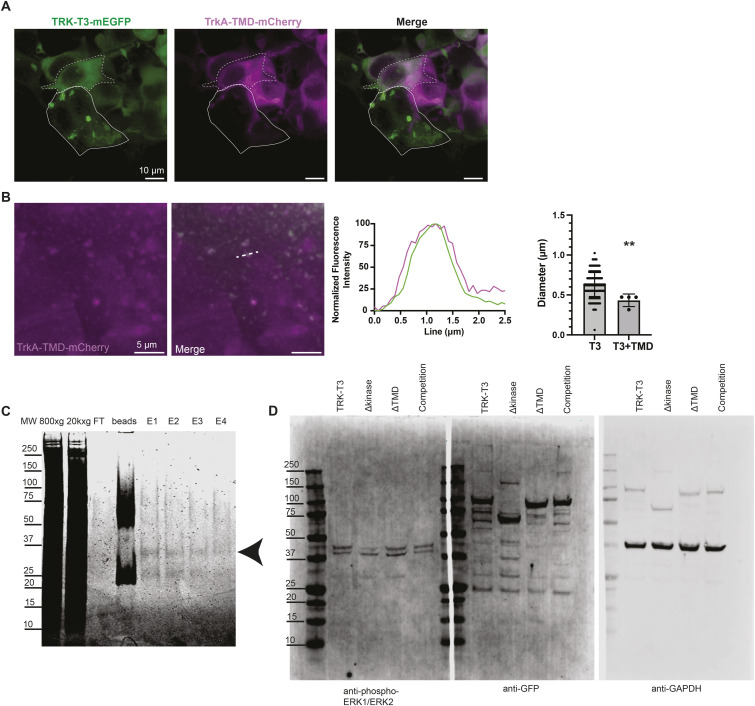
TRK-T3 condensates can be disrupted by a TrkA-TMD mimicking competitor. **(A)** Representative widefield deconvolution micrographs of HEK293T cells transfected with 1 μg of of TRK-T3-mEGFP-FLAG and 1 μg of TrkA-TMD-mCherry-FLAG. Scale bar: 10 μm. Cells with condensates outlined in solid white lines, cells with only reticular pattern outlined in dotted white lines. **(B)** Confocal micrographs in red channel, and merge, corresponding to Fig 5C. Scale bar: 5 μm. Corresponding line scan of TRK-T3-mEGFP-FLAG condensate colocalized with TrkA-TMD-mCherry-FLAG. Graph of condensate diameter of TRK-T3 with or without TrkA-TMD. TRK-T3 condensates, n = 95, TRK-T3 + TrkA-TMD condensates, n = 4, *P* = 0.0087. *P*-values calculated by Welch’s two-tailed *t* test. **(C)** Representative purification overview of TrkA-TMD-mCherry-FLAG with Coomassie stain. **(D)** Representative Western blots for anti-p-ERK (top), anti-GFP (middle), and anti-GAPDH (bottom) from Fig 5D. Source data are available for this figure.

The continued development of drug resistance within cancers necessitates the development of new treatments. Recently, it has been shown that biomolecular condensates can be effective therapeutic targets for disease models (Choi et al, 2025). We set out to test whether TRK-T3 condensates could be disrupted by the addition of a short hydrophobic segment composed of only the hydrophobic TrkA-TMD sequence (Fig 5A). Given the identified importance of the TMD encoded in the TrkA moiety in conferring morphology to TRK-T3 condensates and driving growth signaling, we hypothesized that by exposing full-length TRK-T3 to a competitor TMD segment, the interaction between TRK-T3 molecules might be disrupted. We co-transfected HEK293T cells with full-length TRK-T3-mEGFP-FLAG and TMD-mCherry-FLAG (Figs 5B and S5A). Strikingly, we find that when cells express both TRK-T3 and the hydrophobic TrkA-TMD segment, there is a dose-dependent change in TRK-T3 fluorescence intensity in the absence of condensates (Fig 5B, right graph, open circles). At the lowest expression levels of the TrkA-TMD segment, TRK-T3 forms condensates (filled circles). In addition, we find that when we transfect TrkA-TMD-mCherry into cells expressing TRK-T3-mEGFP, this results in a significant reduction in MAPK activation as measured by phospho-ERK signal (Figs 5D and S5D), indicating that the TMD acted as a competitor to block TRK-T3-mediated signaling. Cells expressing both constructs exhibited a 32% reduction in phospho-ERK signal compared with TRK-T3 alone, when normalized to TRK-T3 expression.

Next, to elucidate whether the ability of the TrkA-TMD segment to disrupt the oncogenic TRK-T3 condensates was dependent on other cytosolic factors, and to exclude putative cell responses contributing to the dissolution of condensates, we established purification of both recombinant TRK-T3-mEGFP-FLAG and TrkA-TMD-mCherry-FLAG. We find that in the presence of the TrkA-TMD segment, TRK-T3 condensates exhibit a significant decrease in the size of condensates formed (Figs 5C and S5B and C). TRK-T3 alone forms condensates of ∼600 nm, n = 95 condensates. Strikingly, in the presence of TrkA-TMD, both the amount of condensates and their diameters were significantly reduced, which was also captured by a significant decrease in the coefficient of variation (Figs 5C and S5B, graph). This reduction in the size of condensates suggests that increasing amounts of TrkA-TMD competitively inhibit the hydrophobic attractive interactions between the TRK-T3 molecules that drive condensate growth. Although enrichment of the competitor TMD was not detected in cells because of their high level of expression and distribution across the cytosol, in vitro, the TMD segment appeared strongly enriched in remnants of TRK-T3 foci (Figs 5C and S5B Merge and line scan). Our findings suggest that the interaction between TrkA-TMD and TRK-T3 is sufficient to disrupt condensate formation and may represent a potential therapeutic avenue for treating cancers that contain the TRK-T3 oncogenic fusion protein.

In conclusion, we propose a model wherein TRK-T3 molecules spontaneously align themselves via contributions of both the TFG and TrkA moieties, particularly driven by the TrkA-TMD to form sheet-like condensates, resembling the oligomerization and planar alignment of RTKs embedded in the PM upon binding to growth factors (Fig 5E). This alignment ensures an efficient autophosphorylation of the kinase domains of the oncogenic fusion protein. Condensates that are formed in proximity to the PM are then able to recruit signal-transduction molecules, such as GRB2, SOS1, and the PM-localized Ras. This sequestration of signal-transduction molecules can subsequently activate key proliferation pathways, such as the MAPK pathway, by bypassing the extracellular cues that ultimately lead to cell transformation (Greco et al, 1995, 1998; Roccato et al, 2002).

## Discussion

Our findings suggest a condensate-based mechanism for TRK-T3-driven oncogenesis. We find that TRK-T3 assembles into sheet-like condensates that form in the cytosol and accumulate near the PM. The source of the TRK-T3 condensate anisotropy and morphology is not yet fully resolved and could reflect coupling to PM-associated factors, such as Ras, or intrinsic molecular alignment within the condensate dense phase. Given the high fluidity of TRK-T3, a planar arrangement would be highly unusual for a liquid-like condensate, whose surface tension is expected to favor spherical droplets. Our data suggest that contributions from both the TFG moiety and the TMD segment promote anisotropy within the condensate dense phase before stable engagement with PM-associated signaling components, consistent with the requirement for kinase autophosphorylation before recruitment of downstream signal-transduction molecules. This organization and localization provide a direct route for kinase domain autophosphorylation and for the efficient recruitment of signal-transduction molecules, including GRB2, mimicking the organization of ligand-activated RTKs in the PM. In addition, we find that TRK-T3 colocalizes well with the ER, suggesting that TRK-T3 could be prewetting the surfaces of the ER membrane, thus increasing TRK-T3 condensation locally, putatively favoring condensation as reported for other proteins (Bagheri et al, 2026; Hoffmann et al, 2026). Given the lack of a signal sequence in TRK-T3, it is unlikely that it is inserted into the ER membrane as an integral membrane protein. It is tempting to speculate that such ER-templated condensates may enrich towards the PM because of a recruitment of signal-transduction molecules such as GRB2 and SOS1, which in turn engage with PM-localized Ras, putatively providing a mechanistic solution to how a cytosolic RTK fusion protein can activate the MAPK pathway independently of receptor insertion into the PM (Fig 5E).

The morphology of TRK-T3 condensates depends on a hydrophobic segment encoded in the TrkA portion of the fusion. Removing this segment eliminates condensate morphology and ER association, abolishes GRB2 recruitment, and diminishes MAPK activation. This role of the TMD contrasts with previous models in which the TFG moiety was proposed to act mainly as an ERES-targeting signal, a mechanism that may still contribute to transformation in other TFG-containing fusion proteins. Instead, our data indicate that contributions from both moieties are required. Although the transmembrane segment appears to be the primary determinant of condensate morphology, the TFG moiety, particularly via the PB1 domain, may contribute to the organization of TRK-T3 within the condensate, thereby enhancing signaling output. In this view, TFG functions as a scaffold that stabilizes molecular alignment within the condensate dense phase.

TRK-T3 is one of several TFG-containing oncogenic fusions. TFG-containing fusions have been detected in acute myeloid leukemia, anaplastic large cell lymphoma, and spindle tumors (Chen & Tseng, 2014), with reported fusions including the RTKs ALK, FGFR1, and RET (Hernández et al, 1999; Loong et al, 2020; Wang et al, 2020). Our findings raise the possibility that condensate geometry, rather than condensate formation alone, may act as an important determinant of signaling potency across this class of fusion proteins. Further analysis of how TFG contributes to the physical organization of additional fusions may reveal broader principles linking condensate architecture to oncogenic signaling strength.

Furthermore, we find that TRK-T3 condensates can be disrupted by adding a short hydrophobic segment that mimics the TrkA transmembrane domain. This dissolution occurs both in cells and in vitro, where the competitor segment interferes with condensate formation. These observations confirm that hydrophobic interactions mediated by the transmembrane domain contribute directly to TRK-T3 condensate organization. Because kinase inhibitors are often associated with resistance, targeting the physical organization of the fusion protein itself may offer an orthogonal intervention point. Elucidating condensation mechanisms across additional TFG-containing fusions may inform whether similar principles can be leveraged to develop micropeptide-based “kill switches” (Deng et al, 2025; Zhang et al, 2025) as interventions.

## Materials and Methods

### Cell culture/transfection/labeling

HeLa cells (CCL2; ATCC) and HEK293T (3216; ATCC) cells were grown at 37°C at 5% CO_2_ in Dulbecco’s modified eagle medium (cat #10566-016; Gibco) supplemented with 10% FBS [A31604-01, A5669402; Gibco]. Cells were seeded on plain glass-bottom or collagen-coated glass-bottom Mattek dishes (P35GC-1.5-14-C, P35GCOL-1.5-14-C) for imaging. For transient overexpression, cells were transfected with TRK-T3-mEGFP-FLAG, TRK-T3-SNAP-FLAG, TRK-T3-mCherry-FLAG, TRK-T3-mEGFP-FLAG domain deletion variants, KDEL-Halo and/or TrkA-TMD-mCherry-FLAG at 1 μg for HEK293T cells. TRK-T3-mEGFP-FLAG/TRK-T3-SNAP-FLAG and domain deletion variants were transfected in HeLa cells at 2 μg and GRB2-EGFP at 0.5 μg and left to express for 24 h. Transfections were performed using Fugene transfection reagent (cat #E2311; Promega). For PM localization, HEK293T cells transfected with TRK-T3-mEGFP-FLAG were stained with Invitrogen CellLight Plasma Membrane-RFP, BacMam 2.0 (cat# C10608) 8 h after transfection at 30 particles per cell. For SNAP and Halo tagged constructs, 1 μM of ligand was used. For SNAP, HEK cells were incubated with 1 ml of μM solution in DMEM (cat #S9105S; SNAP-Cell 647-SiR, cat #S9102S; SNAP-Cell TMR-Star) for 30 min, rinsed, and then imaged; HeLa cells were incubated for 30 min, rinsed, then incubated for 30 min in fresh DMEM before imaging. For HEK cells stained with Halo ligand (cat #G825B; HaloTag TMR Ligand), cells were stained for 15 min, washed, then incubated for 1 h with fresh DMEM before imaging.

For immunofluorescence studies, cells were fixed with 4% PFA (cat # 50980487; Electron Microscopy Sciences) for 15 min. Fixed cells were washed with PBS and permeabilized with permeabilization buffer (0.3% IGEPAL, 0.05% Triton-X 100, 0.1% BSA), then washed with wash buffer (0.05% IGEPAL, 0.05% Triton-X 100, 0.2% BSA), and blocked with blocking buffer (0.05% IGEPAL, 0.05% Triton-X 100, 5.0% normal goat serum) for 1 h at room temperature. As indicated, cells were labeled with antibodies diluted in blocking buffer, anti-SEC16 at 1:200 (cat #PA552182; Invitrogen) or anti-Tango1 at 1:1,000 (cat #ab244506; Abcam) for 1 h at room temperature or overnight at 4°C, washed, and labeled with goat-anti-mouse (cat #A32727; Invitrogen) or goat-anti-rabbit (cat #A32732; Invitrogen) Alexa Fluor Plus, conjugated secondary antibodies at 1:1,000 for 1 h, subsequently washed and imaged.

Immunoblotting was carried out as follows: six-well plates were transfected with 1 μm/well of mEGFP-FLAG plasmid, TRK-T3-mEGFP-FLAG, TRK-T3ΔPB1-mEGFP-FLAG, TRK-T3ΔTMD-mEGFP-FLAG, or TRK-T3Δkinase-mEGFP-FLAG. Cells were allowed to express for 24 h. Before collecting, untransfected wells were treated with 1 ng/ml of epidermal growth fact (PHG0311L Human EGF Recombinant Protein: Thermo Fisher Scientific) for 7.5 min. Cells were rinsed with PBS and then scraped with lysis buffer (50 mM Hepes/KOH, pH 7.3, 150 mM KCl, EDTA-free protease inhibitor cocktail tablet [Roche], phosphatase inhibitor tablet [PhosSTOP Roche], and 0.1% Triton-X 100). Cell lysates were boiled and run on 4–20% Bis-Tris gradient gel. Western blotting was performed using Thermo Fisher Scientific anti-GFP antibody at 1:2,000 (cat #A11122), Abcam anti-phospho-ERK antibody at 1:1,000 (cat #ab201015), Abcam anti-GAPDH antibody at 1:10,000 (cat #ab8245), Abcam anti-TFG at 1:2,500 (cat #156866) and Sigma-Aldrich anti-FLAG at 1:2,500 (cat #F1804). Heterozygous HeLa TFG-mClover::FLAG cells were transfected with 50 ng, 100 ng, 250 ng, 500 ng, 1 μg, or 2 μg of TRK-T3-mEGFP-FLAG per well. Western blots were performed as indicated above.

### Endogenous tagging of TFG in HeLa cells

Homozygous TFG::mClover-FLAG knock-in HeLa cells were generated by ExpressCells (ExpressCells, Inc.) via CRISPR/Cas9 and validated by ExpressCells via PCR and Sanger sequencing. In addition, the cell line was validated via Western blotting (Abcam anti-TFG at 1:2,500 [cat #156866]) and fluorescence microscopy as reported previously (Bogus et al, 2025).

### Plasmid sequences

The sequences for plasmids used were sourced from GenBank at NCBI and included TRK-T3 X85960.1. The TRK-T3 and domain deletion constructs were generated via commercial gene synthesis (gBLOCK; IDT). gBLOCKs and the pSNAPf plasmid (N9183S; NEB) or the pmEGFP-FLAG and pmCherry-FLAG previously described (Bogus et al, 2025) were both digested with NheI (R0189S; NEB) and AgeI (R3552S; NEB) restriction enzymes to insert the commercially obtained gene via ligation with T4 ligase (10481220001; Sigma-Aldrich) as per the manufacturer’s instructions.

The ligation mixture was transformed into Subcloning Efficiency DH5alpha competent cells (18265017; Thermo Fisher Scientific), plated onto 100 μg/ml ampicillin agar plates (LA-2100; Biomyx), and several single colonies were cultured in LB and 100 μg/ml ampicillin. Single colony-derived cultures were subsequently mini-prepped using a Qiaprep Spin Miniprep Kit (27104; QIAGEN), and plasmid sequences were confirmed via sequencing by Azenta/GeneWiz. TrkA-TMD-mCherry-FLAG was generated by creating primer sequences 5′-CTA​GCA​TGA​CAC​CTT​TTG​GGG​TCT​CGG​TGG​CTG​TGG​GCC​TGG​CCG​TCT​TTG​CCT​GCC​TCT​TCC​TTT​CTA​CGC​TGC​TCC​TTG​TGC​TCA-3′ and 5′-CCG​GTG​AGC​ACA​AGG​AGC​AGC​GTA​GAA​AGG​AAG​AGG​CAG​GCA​AAG​ACG​GCC​AGG​CCC​ACA​GCC​ACC​GAG​ACC​CCA​AAA​GGT​GTC​ATG-3′ and allowing them to anneal in 1× Cloned Pfu DNA polymerase reaction buffer (Catalog #600250) at 75°C for 60 min, then overnight at 4°C. The mixture created a double-stranded sequence with overhangs matching cut NheI and AgeI sites that was then incubated with NheI and AgeI-digested pmCherry-FLAG plasmid. TRK-T3-aH1 constructs were generated by creating primer sequences 5′-GAA​CCA​CCT​GGA​GAA​CCA​GGA-3′ and 5′-TTC​AAG​GGG​TCT​TGG​CTG​G-3′ and using the Q5 site-directed mutagenesis kit as per the manufacturer’s instructions (E0554S; NEB). Sequences were confirmed as above. GRB2-EGFP plasmid was obtained from Addgene (Plasmid #86873). TFG(1–193)-mEGFP-FLAG and TrkA(399–796)-mEGFP-FLAG were manufactured by Twist Bioscience.

### Microscopy

Conventional (widefield/deconvolution, confocal) microscopy was performed on a Cytiva OMX SR microscope setup equipped with an Olympus PlanApo N 60X/1.42 oil objective. Multichannel alignment was performed by the manufacturer using TetraSpeck beads. Multicolor micrographs were aligned using OMX-specific software packages (softWoRx). Images of immunolabeled cells were obtained using widefield with deconvolution, or confocal microscopy. Excitation for green channel: 488 nm, red channel: 568 nm, far red channel: 640 nm. Emission filters for green channel: 500–550, red channel: 609–654, far red channel: 665–705. For time-lapse imaging of transfected cells, images at 37°C were acquired once every 15 min for 8–12 h using conventional widefield or confocal mode. Images were processed by using OMX-specific software packages (softWoRx). Size measurements were made in ImageJ, and plots and statistical measurements were made in GraphPad Prism.

For live cell imaging with live imaging solution (A59688DJ; Invitrogen) supplemented with 10 mM final concentration of glucose (G8644-100Ml; Sigma-Aldrich).

Colocalization experiments were analyzed using the ImageJ plugin BIOP (Bioimaging and Optics Platform) JACoP using Otsu’s method for automatic thresholding to calculate Pearson’s coefficient.

For FRAP analysis of TRK-T3 condensates, cells were transfected as described above and individual TRK-T3 condensates from n > 3 cells were partially or fully bleached using the FRAP module in the OMX SR platform combined with confocal mode at 37°C. Recovery was measured using the Time Series Analyzer V3 plugin in ImageJ, and plots were generated using GraphPad Prism.

All in vitro confocal and TIRF images were taken with the sample spotted on plain glass-bottom dishes and at room temperature unless otherwise indicated (P35G-1.5-14-C; Mattek).

### Protein purification from Expi293 cells

Full-length and truncated TRK-T3-encoding, as well as TrkA-TMD-mCherry, plasmids were transfected into Expi293F cells (Thermo Fisher Scientific) at 1 μg/ml culture using Expifectamine 293 reagent as per the manufacturer’s instructions. Time-course experiments were conducted to ensure optimal expression time for constructs, with expression times varying from 16 to 72 h. Expi293F cells were incubated at 37°C, 8% CO_2_ on a ThermoFisher Scientific shaker (cat no. 88881101) at 120 rpm (orbit diameter: 19 mm). Cells were pelleted at 800*g* for 10 min and frozen in liquid nitrogen. Pellets between 30 and 60 ml of culture, depending on the construct, were resuspended in 10 ml of buffer (50 mM Hepes/KOH, pH 7.3, 150 mM KCl, EDTA-free protease inhibitor cocktail tablet [Roche]). Cells were then homogenized using 20 passages through a 25G needle. Lysate was centrifuged at 800*g* for 30 min. Pelleted material was discarded, and supernatant was centrifuged at 20,000*g* for 15 min. Pelleted material was discarded, and the supernatant was transferred to a conical tube containing 2.5 ml blocked FLAG-affinity resin equilibrated with buffer (50 mM Hepes/KOH, pH 7.3, 500 mM KCl, EDTA-free protease inhibitor tablet [Roche]) and rotated for 1 h at room temperature. The suspension was settled on a column that was equilibrated with 50 ml of buffer. The resin was then washed with 100 ml of buffer. Next, the column was drained, and 1 ml of ATP wash buffer (buffer plus 0.5 mM ATP, 0.5 mM MgCl2 [Thermo Fisher Scientific]) was added to the resin and incubated for 10 min, followed by a wash of 50 ml buffer without ATP. The proteins were eluted in elution buffer (buffer plus 200 μM FLAG peptide [Sigma-Aldrich]) for 1.25 h, yielding 2–5 fractions total. Elutions were analyzed on 4–20% Bis-Tris gradient gels, stained with Coomassie, and analyzed on a LI-COR Odyssey infrared scanner. After elution, fractions were frozen in liquid nitrogen before further processing. Western blotting of TRK-T3-mEGFP-FLAG was performed using Thermo Fisher Scientific anti-GFP antibody (cat# A11122).

Concentration of samples was performed using a 50 kD Amicon Ultra-0.5 Centrifugal Filter Unit (UFC5050; Millipore) for the TRK-T3-mEGFP-FLAG variants and a 3 kD Amicon unit for the TrkA-TMD-mCherry construct. The elution was thawed at 37°C with occasional vortexing, and the sample was centrifuged at 21.1*g* for 10 min before concentration. The column was washed in 200 μl steps with 2 ml total of 50 mM Hepes/KOH, pH 7.3, and 150 mM KCl. Next, the sample was concentrated further stepwise in the Amicon column, and the sample was used immediately for experiments without further storage. Sessile droplet evaporation was performed at room temperature after concentration in 50 mM Hepes/KOH, pH 7.3, and 150 mM KCl.

TRK-T3 protein model (Fig 5E) was created by AlphaFold3 (Abramson et al, 2024).

## Supplementary Material

Reviewer comments

## Data Availability

Source data are available below. All other data are available from the corresponding authors upon reasonable request.
