# Supplementary material for: TRK-T3 condensate organization drives growth signaling
Source: Life Sci Alliance. 2026 Jun 17;9(9):e202603653. doi: 10.26508/lsa.202603653 (PMC13276355; doi:10.26508/lsa.202603653)
Supplement: Reviewer comments [file LSA-2026-03653_review_history.pdf]

TRK-T3 Condensate Organization Drives Growth Signaling

Sindy Chavez, Savannah Bogus, Samantha Cheung, Winnie Zhou, Lilja Gudmundsson, Kaho Kato, and Andreas Ernst
DOI: https://doi.org/10.26508/lsa.202603653

Corresponding author(s): Andreas Ernst, University of California San Diego

Review Timeline:

Submission Date:	2026-02-02
Editorial Decision:	2026-03-10
Revision Received:	2026-04-23
Editorial Decision:	2026-05-22
Revision Received:	2026-06-01
Accepted:	2026-06-05

Scientific Editor: Sarita Hebbar

Transaction Report:

(Note: With the exception of the correction of typographical or spelling errors that could be a source of ambiguity, letters and reports are not edited. The original formatting of letters and referee reports may not be reflected in this compilation.)

March 10, 2026

Re: Life Science Alliance manuscript #LSA-2026-03653-T

Prof. Andreas M Ernst
University of California San Diego
School of Biological Sciences; Cell & Developmental Biology
9500 Gilman Dr
La Jolla 92093

Dear Dr. Ernst,

Thank you for submitting your manuscript entitled "TRK-T3 Condensate Geometry Drives Growth Signaling" to Life Science Alliance.
Your manuscript was assessed by three reviewers whose comments are appended below. As you will read, two out of three reviewers are supportive of your study and its value. That said, all the reviewers raised significant concerns that preclude publication of this work for now. In line with the overall evaluation of Reviewer 1 and 2, we invite you to submit a revised manuscript addressing their concerns.

We agree with the reviewers that you must provide evidence if the endogenous TRK-T3 forms similar condensates to rule out the effects of protein overexpression (Reviewer 1 - Figure 1B and Reviewer 3 - point 2.1a). Next, in terms of the TRK-T3 condensates accumulation at the plasma membrane we point you to the concerns of concerns of all the three reviewers ((Reviewer 1, point 2, Reviewer 2, point 3, Reviewer 3, point 2.3 and Figure 4B). These points are related to data analyses (Reviewer 3) which we agree must be addressed. We also agree that you must either provide appropriate evidence or you must explicitly include the possibility that condensate co-localisation in ER is not ruled out. You must expand the discussion along the lines of the comment from Reviewer 2 on membrane prewetting when discussing the accumulation in the vicinity of the plasma membrane.
Finally we concur with Reviewers 2 and 3 that a more comprehensive in-vitro characterisation of LLPS of Trk-T3 must be provided to differentiate it from aggregation (Reviewer 2, point 4 and Reviewer 3, point 2.1c and Figure 3D).

When submitting the revision, please include a letter addressing the reviewers' comments point by point. While a rebuttal must respond to all points in some form, additional experiments to resolve these points, other than indicated above, are not required.

I would be happy to discuss the revision in more detail via email or phone/videoconferencing. Please let me know which option you prefer, if any.

To upload the revised version of your manuscript, please log in to your account: https://lsa.msubmit.net/cgi-bin/main.plex

You will be guided to complete the submission of your revised manuscript and to fill in all necessary information. Please get in touch in case you do not know or remember your login name.

The typical timeframe for revisions is three months. Please note that papers are generally considered through only one revision cycle, so strong support from the referees on the revised version is needed for acceptance.

While you are revising your manuscript, please also attend to the below editorial points to help expedite the publication of your manuscript. Please direct any editorial questions to the journal office.

Thank you for this interesting contribution to Life Science Alliance. We hope that the comments below will prove constructive as your work progresses, and we are looking forward to receiving your revised manuscript.

Sincerely,

Sarita Hebbar, PhD
Scientific Editor
Life Science Alliance
http://www.lsajournal.org

A. THESE ITEMS ARE REQUIRED FOR REVISIONS

-- A letter addressing the reviewers' comments point by point.

-- An editable version of the final text (.DOC or .DOCX) is needed for copyediting (no PDFs).

-- High-resolution figure, supplementary figure and video files uploaded as individual files: See our detailed guidelines for preparing your production-ready images, https://www.life-science-alliance.org/authors

-- Summary blurb (enter in submission system): A short text summarizing in a single sentence the study (max. 200 characters including spaces). This text is used in conjunction with the titles of papers, hence should be informative and complementary to the title and running title. It should describe the context and significance of the findings for a general readership; it should be written in the present tense and refer to the work in the third person. Author names should not be mentioned.

-- By submitting a revision, you attest that you are aware of our payment policies found here: https://www.life-science-alliance.org/copyright-license-fee

B. MANUSCRIPT ORGANIZATION AND FORMATTING:

Full guidelines are available on our Instructions for Authors page, https://www.life-science-alliance.org/authors

We encourage our authors to provide original source data, particularly uncropped/-processed electrophoretic blots and spreadsheets for the main figures of the manuscript. If you would like to add source data, we would welcome one PDF/Excel-file per figure for this information. These files will be linked as supplementary "Source Data" files.

IMPORTANT: It is Life Science Alliance policy that if requested, original data images must be made available. Failure to provide original images upon request will result in unavoidable delays in publication. Please ensure that you have access to all original microscopy and blot data images before submitting your revision.

Reviewer #1 (Comments to the Authors (Required)):

In this study, the authors report that the TFG-TrkA fusion forms functional biomolecular condensates. These condensates are localized in proximity to the plasma membrane, where they recruit downstream signaling components and activate downstream signaling pathways. The authors further design a peptide-based competitive strategy to disrupt the formation of these condensates.

The manuscript demonstrates a solid understanding of the relevant literature and reports several interesting observations. However, the overall quality of the study requires further improvement. The following concerns need to be addressed to strengthen the manuscript.
Major comments:
1. Line 129: The respective contributions of TFG[1-193] and TrkA[399-796] to condensate formation should be systematically evaluated, for example by employing a well-established optodroplet-based assay.
2. Line 133: Co-localization experiments with endoplasmic reticulum markers (e.g., calnexin or Sec61β) need to be performed to rule out the possibility that TRK-T3 co-localizes with the ER.
3. In Fig. 5, more functional experiments showing that disruption of condensate formation leads to loss of downstream MAPK pathway activation need to be added. e.g., Disruption of hydrophobic interactions within the condensates abolishes pERK activation and prevents the recruitment of downstream signaling molecules.

Minor comments:
1. Fig. 1B，experiments using lower TRK-T3 expression levels should be added to exclude the possibility that the observed effects are driven by excessive protein overexpression.
2. Fig. 3D and Fig. 5C lack descriptions of protein concentration, buffer composition, and experimental temperature about the sessile droplet evaporation assay. Please provide these details in the figure legend or the Methods section.
3. In Fig. 4C, the Western blot lacks the mEGFP control lane, although it is included in the adjacent quantification bar graph. Please ensure consistency. In addition, the ERK blot is lacked and the GAPDH blot in Supp.Fig4C should be included.

Reviewer #2 (Comments to the Authors (Required)):

In the submitted manuscript, Chavez, Bogus et al showed that TRK-T3, a TFG-TrkA fusion oncoprotein, forms planar, sheet-like biomolecular condensates whose geometry is essential for MAPK pathway activation. Using transient overexpression in HEK293T and HeLa cells combined with widefield/confocal imaging, time-lapse microscopy, and FRAP, they demonstrate that

cytosolic TRK-T3 assembles into fluid, planar condensates that accumulate near the plasma membrane yet remain distinct from ER-exit-site markers. Domain deletions and quantitative analysis of condensate number, size, and coefficient of variation reveal that a hydrophobic TrkA transmembrane segment is required to impose planar geometry both in cells and in vitro. Co-expression with GRB2 and GRB2 colocalization analysis, together with phospho-ERK immunoblotting of TRK-T3 variants, show that this geometry promotes kinase autophosphorylation, GRB2 recruitment, and robust MAPK activation, whereas loss of the TMD or kinase domain diminishes signaling. Finally, a minimal peptide mimicking the TrkA TMD competitively disrupts TRK-T3 condensates, highlighting condensate geometry and TMD-mediated hydrophobic interactions as a potential therapeutic vulnerability. Overall, this is a very timely study focusing on an emerging topic of condensate-membrane interactions, and I strongly endorse the publication of these data once the following aspects are addressed.

1. To separate structural condensate effects from catalytic output, the authors should test kinase-dead TRK-T3 and phospho-site mutants verifying that condensate geometry remains intact but GRB2 recruitment and pERK elevation are abolished.

2. Are the condensate formation vs. pathway output differentially disrupted? What happens upon combining TRK-T3 expression with Ras inhibition, MEK/ERK inhibitors, or dominant-negative Ras?

3. Thematically, the authors should directly relate discriminate presetting from bulk condensation at the membrane. Are TRK-T3 "planar condensates" in fact pre-existing, membrane-coupled enrichment layers or bona fide bulk LLPS, explicitly relating the observed behavior to recent work on membrane prewetting and phase separation the Veatch and Milovanovic labs (PMID: 41482580 and PMID: 41512824).

4. The in-vitro experiments are valuable in dissecting the mechanism. The authors should systematically vary salt, crowding, and TMD peptide stoichiometry for purified TRK-T3 and TRK-T3ΔTMD to map phase diagrams and demonstrate that planar condensates and signaling correlate with a distinct LLPS regime, not generic aggregation.

Reviewer #3 (Comments to the Authors (Required)):

This study examines how the oncogenic fusion protein TRK-T3 activates MAPK signalling by forming planar, sheet-like condensates in the cytosol, distinct from the spherical condensates formed by TFG alone.

The study shows that the condensate geometry is driven by a hydrophobic transmembrane domain (TMD) derived from TrkA. Deletion of the TMD disrupts planar condensate formation and significantly reduces recruitment of GRB2 and downstream ERK phosphorylation. While other domains contribute to signaling, the TMD is the primary determinant of condensate architecture and signaling strength. The data suggest that planar condensates spatially organize kinase domains to promote efficient autophosphorylation, functionally mimicking ligand-induced RTK clustering. A minimal hydrophobic peptide corresponding to the TMD disrupts condensate formation and attenuates signaling, highlighting hydrophobic interactions as a potential therapeutic target.

The paper has major shortcomings which are listed below, the work is very preliminary and needs many validations and would be unsuitable to publish if it cannot answer the following concerns

2. Main points of the paper and major concerns
2.1 TRK-T3 form planar, sheet-like condensate
Concern 2.1.a- What is the endogeneous concentration of TRK-T3 in cells? Do the endogenous proteins form similar condensates ?
Concern 2.1.b-Localisation of the condensates is not very clear. The authors should conduct additional confirmatory experiments to demonstrate that these condensates form on the inner side of the membrane. E.g. if the membrane is disturbed, will the condensates also get disturbed ?
Concern 2.1.c- In vitro condensate assays shown in Figure 3D are based on the progressive drying method. This method leads to an increase in concentration of the protein and salt as the water evaporates. This is a very poor experiment to show phase separation as we do not know the final concentration and salt concentration at which the phase separation takes place.
Concern 2.1.4- The material properties of the in vitro condesate is unclear as no FRAp experiments are shown.
2.2 TMD is necessary for the planar geometry of condensates

2.3 TRK-T3 condensates localize near the plasma membrane
The authors showed that the TRK-T3 forms planar sheet like condesate, and suggest that these condesates are formed on the underside of the plasma membrane.
Concern 2.3.a- Its not really clear if they are really on the underside of the membrane

Concern 2.3.b- FRET experiments should be shown between membrane inner side protein and TRK-T3
2.4 Condensate planar geometry is required for the GRB2 recruitment and MAPK activation
Since this fusion protein was studied to show how MAPK pathway is activated in cancer, a cellular model system for inducing normal cells to cancerous cells due to this type of fusion protein, dependent on condensate internal geometry would be

desirable.
2.5 A minimal TMD peptide can disrupt TRK-T3 condensates
Data strongly support a dose-dependent effect of the hydrophobic TrkA-TMD peptide on condensate dissolution, both in cells and in vitro.
Concerns: The Specificity of the TMD peptide has not been tested.

Quantification concerns

Fig. 1. J - no quantification sof mobile fractions or time scales
Fig. 3D- No quantification of the fraction of protein in the droplet vs outside
Fig. 4B- colocalization coefficients should be calculated, as line profile of 1 foci in 1 cell is cherry picking, and not statistically testable

Response to reviewer critiques

We thank all reviewers for their constructive feedback on our manuscript.

Thank you for submitting your manuscript entitled "TRK-T3 Condensate Geometry Drives Growth Signaling" to Life Science Alliance.

Your manuscript was assessed by three reviewers whose comments are appended below. As you will read, two out of three reviewers are supportive of your study and its value. That said, all the reviewers raised significant concerns that preclude publication of this work for now. In line with the overall evaluation of Reviewer 1 and 2, we invite you to submit a revised manuscript addressing their concerns.

We agree with the reviewers that you must provide evidence if the endogenous TRK-T3 forms similar condensates to rule out the effects of protein overexpression (Reviewer 1 - Figure 1B and Reviewer 3 - point 2.1a). Next, in terms of the TRK-T3 condensates accumulation at the plasma membrane we point you to the concerns of concerns of all the three reviewers ((Reviewer 1, point 2, Reviewer 2, point 3, Reviewer 3, point 2.3 and Figure 4B). These points are related to data analyses (Reviewer 3) which we agree must be addressed. We also agree that you must either provide appropriate evidence or you must explicitly include the possibility that condensate co-localisation in ER is not ruled out. You must expand the discussion along the lines of the comment from Reviewer 2 on membrane prewetting when discussing the accumulation in the vicinity of the plasma membrane.

Finally we concur with Reviewers 2 and 3 that a more comprehensive in-vitro characterisation of LLPS of Trk-T3 must be provided to differentiate it from aggregation (Reviewer 2, point 4 and Reviewer 3, point 2.1c and Figure 3D).

Previous studies investigating TRK-T3 have employed overexpression systems to address underlying questions about this protein. In the original study that identified TRK-T3, Greco et al (1995) describe overexpression of TRK-T3 in both HeLa and 3T3 cells to study TRK-T3's localization and transformation capabilities. Witte et al (2011) also used an overexpression system to investigate the localization of TRK-T3 with endoplasmic reticulum exit site marker Sec16 and its ability to activate the MAPK pathway. In Zhu et al (2023), several NTRK1 fusions, including a version of TRK-T3, were studied by stably expressing these fusions and studying their propensity to form biomolecular condensates in HEK293T and 3T3 cells.

To address this concern, we overexpressed TRK-T3-mEGFP-FLAG in HeLa cells in which TFG is endogenously tagged with mClover-FLAG in a heterozygous manner. We compared the expression of TRK-T3-mEGFP-FLAG to that of TFG::mClover-FLAG via Western blot by using an anti-TFG antibody. We find that the expression of TRK-T3 at 2 µg is 8-fold higher than that of TFG::mClover. Additionally, at 1 µg, where we also observe TRK-T3 condensates, the expression of TRK-T3 is 4-fold higher than that of TFG::mClover. Previously, we have shown that TFG, which makes up the N-terminal portion of TRK-T3, is able to form biomolecular condensates at endogenous levels in cells (1 µM), and even at concentrations 10x below endogenous levels in vitro (Bogus et al., 2025). When TFG is overexpressed, the same basic structures formed by endogenous TFG, i.e. hollow anisotropic condensates of ~300nm, are preserved in the overexpressed system but enlarged. The large TRK-T3 domains we observe are thus likely not due to meeting a saturation concentration that is not met at endogenous levels but rather increases the volume fraction of dense phase in the cell, allowing their visualization using microscopy-based approaches.

Additionally, while it is hypothesized that the TRK-T3 fusion gene is under the control of the TFG promoter in cancer cells harboring this chromosomal translocation, there is no clear evidence that this is the case. Additionally, the genomic instability in cells harboring this translocation would likely change the context of expression, making it difficult to ascertain the exact expression of all cells harboring the TRK-T3 fusion.

We have addressed TRK-T3 colocalization concerns for each reviewer. For reviewer 1's concern, we have added a measure of colocalization of TRK-T3 with a general marker for the endoplasmic reticulum (Fig. 2 C and Supp. Fig. 2 C). Reviewer 2's comment on membrane prewetting has been addressed in the discussion (lines 379 – 385), this concern raises an exciting possibility that TRK-T3 condensates build via pre-wetting. We have addressed reviewer 3's concern by more clearly stating how the colocalization of TRK-T3 and GRB2 was performed (lines 280 – 281) and we revised Figure 4 to more clearly reflect the colocalization.

We thank reviewers 2 and 3 for their concerns and have added more robust in-vitro characterization of TRK-T3 (Fig 3 D) which shows spontaneous phase transition more clearly. Furthermore, we have updated the methods section to better express buffer conditions etc.

Reviewer #1 (Comments to the Authors (Required)):

In this study, the authors report that the TFG-TrkA fusion forms functional biomolecular condensates. These condensates are localized in proximity to the plasma membrane, where they recruit downstream signaling components and activate downstream signaling pathways. The authors further design a peptide-based competitive strategy to disrupt the formation of these condensates.

The manuscript demonstrates a solid understanding of the relevant literature and reports several interesting observations. However, the overall quality of the study requires further improvement. The following concerns need to be addressed to strengthen the manuscript. Major comments:

1. Line 129: The respective contributions of TFG[1-193] and TrkA[399-796] to condensate formation should be systematically evaluated, for example by employing a well-established optodroplet-based assay.

We have established that neither moiety on its own can form condensates (Fig. 1 B i and ii, 1 E i and ii, Supp. Fig. 1 A and 1 B). Additionally, individual domains contributed by each moiety were deleted (Fig. 3 B and Supp. Fig. 3 A and B). We thank the reviewer for the idea of employing optodroplet-based assays, but believe they are outside the scope of this manuscript.

2. Line 133: Co-localization experiments with endoplasmic reticulum markers (e.g., calnexin or Sec61β) need to be performed to rule out the possibility that TRK-T3 co-localizes with the ER.

We thank the reviewer for this suggestion and we have added Fig 2 C. Here, we co-overexpress KDEL-Halo as an ER marker combined with and TRK-T3-mEGFP. We performed confocal microscopy and colocalization analysis on sum projected images to measure Pearson's correlation. We find that the correlation is high (Pearson's coefficient 0.889 ± 0.084).

3. In Fig. 5, more functional experiments showing that disruption of condensate formation leads to loss of downstream MAPK pathway activation need to be added. e.g., Disruption of hydrophobic interactions within the condensates abolishes pERK activation and prevents the recruitment of downstream signaling molecules.

We thank the reviewer for this suggestion, and we have added Fig 5 D. Here, we show that when we transfect TrkA-TMD-mCherry into cells expressing TRK-T3-mEGFP there is an approximately 30% reduction in phosphorylated-ERK expression compared to only full-length TRK-T3.

Minor comments:

1. Fig. 1B, experiments using lower TRK-T3 expression levels should be added to exclude the possibility that the observed effects are driven by excessive protein overexpression.

We agree with the reviewer's suggestion and added Supplemental Figure 1 H and I. In Supp. Fig 1 H, we compare the expression levels of TRK-T3-mEGFP-FLAG at increasing concentrations in HeLa cells that are endogenously tagged at the TFG locus with mClover-FLAG. We find that at 2 μg, there is an 8-fold increase in TRK-T3 expression compared to endogenous TFG expression. However, even at the lowest concentration transfected, 50 ng, there is a 4-fold increase. Additionally, we analyzed the expression of HeLa cells transfected with 2 μg of TRK-T3-mEGFP-FLAG and find that at lower protein expression, as measured by total cell integrated intensity divided by cell area, evidence of TRK-T3 condensation is observed (Supp. Fig. 1 I iii).

2. Fig. 3D and Fig. 5C lack descriptions of protein concentration, buffer composition, and experimental temperature about the sessile droplet evaporation assay. Please provide these details in the figure legend or the Methods section.

We thank the reviewer for this suggestion and have updated the methods section to more clearly state these parameters. (line 672 – 673).

3. In Fig. 4C, the Western blot lacks the mEGFP control lane, although it is included in the adjacent quantification bar graph. Please ensure consistency. In addition, the ERK blot is lacked and the GAPDH blot in Supp.Fig4C should be included.

We thank the reviewer for this suggestion and have updated Figure 4 C.

Reviewer #2 (Comments to the Authors (Required)):

In the submitted manuscript, Chavez, Bogus et al showed that TRK-T3, a TFG-TrkA fusion oncoprotein, forms planar, sheet-like biomolecular condensates whose geometry is essential for MAPK pathway activation. Using transient overexpression in HEK293T and HeLa cells combined with widefield/confocal imaging, time-lapse microscopy, and FRAP, they demonstrate that cytosolic TRK-T3 assembles into fluid, planar condensates that accumulate near the plasma membrane yet remain distinct from ER-exit-site markers. Domain deletions and quantitative analysis of condensate number, size, and coefficient of variation reveal that a hydrophobic TrkA transmembrane segment is required to impose planar geometry both in cells and in vitro. Co-expression with GRB2 and GRB2 colocalization analysis, together with phospho-ERK immunoblotting of TRK-T3 variants, show that this geometry promotes kinase autophosphorylation, GRB2 recruitment, and robust MAPK activation, whereas loss of the TMD or kinase domain diminishes signaling. Finally, a minimal peptide mimicking the TrkA TMD competitively disrupts TRK-T3 condensates, highlighting condensate geometry and TMD-mediated hydrophobic interactions as a potential therapeutic vulnerability. Overall, this is a very timely study focusing on an emerging topic of condensate-membrane interactions, and I strongly endorse the publication of these data once the following aspects are addressed.

1. To separate structural condensate effects from catalytic output, the authors should test kinase-dead TRK-T3 and phospho-site mutants verifying that condensate geometry remains intact but GRB2 recruitment and pERK elevation are abolished.

We agree with the reviewer's suggestion and provide this data in Figures 3 and 4. In Figure 3, we show that TRK-T3Δkinase still forms condensates without a change in geometry as compared to full-length TRK-T3. In figure 4 B and supplemental figure 4 B, we show that TRK-T3Δkinase can no longer bind GRB2, and in Figure 4 C, we show that TRK-T3Δkinase does not exhibit elevated phospho-ERK expression compared to full-length TRK-T3. We believe that additional experiments involving individual phospho-site mutants are not in the scope of this study.

2. Are the condensate formation vs. pathway output differentially disrupted? What happens upon combining TRK-T3 expression with Ras inhibition, MEK/ERK inhibitors, or dominant-negative Ras?

We thank the reviewer for these intriguing suggestions but believe that these experiments are outside the scope of this manuscript, which establishes that TRK-T3 condensation is driven by a hydrophobic domain that controls a planar condensate geometry, mediates efficient recruitment of GRB2, and leads to activation of MAPK. In future work, we plan on building on the *in vitro* approaches to characterize the sequential recruitment of signal transduction molecules, for which the reviewer's suggestions are much appreciated.

3. Thematically, the authors should directly relate discriminate presetting from bulk condensation at the membrane. Are TRK-T3 "planar condensates" in fact pre-existing, membrane-coupled enrichment layers or bona fide bulk LLPS, explicitly relating the observed behavior to recent work on membrane prewetting and phase separation the Veatch and Milovanovic labs (PMID: 41482580 and PMID: 41512824).

We thank the reviewer for raising this interesting point of discussion. We have added additional data in Fig. 2 C, where we show that TRK-T3 colocalizes well with a general ER marker (KDEL). These data present the possibility of TRK-T3 condensation being facilitated by the accumulation at the ER, potentially through prewetting at ER membranes rather than through bulk phase separation. How these domains then accumulate at the plasma membrane remains unknown but is an exciting future direction to explore. We have also updated our discussion to include these questions in lines 379 – 385.

4. The in-vitro experiments are valuable in dissecting the mechanism. The authors should systematically vary salt, crowding, and TMD peptide stoichiometry for purified TRK-T3 and TRK-T3ΔTMD to map phase diagrams and demonstrate that planar condensates and signaling correlate with a distinct LLPS regime, not generic aggregation.

We thank the reviewer for these suggestions. We believe it is outside the scope of this manuscript to generate complete phase diagrams, as the fusion protein is difficult to purify at yields enabling such analysis. Our manuscript focuses on the unusual link between the dense phase geometry of TRK-T3 and that this geometry, and subsequent signaling capacity, depends on a specific hydrophobic segment within the fusion protein. We agree with the concern of putative aggregation in vitro, and have added Figure 3 D, where we present evidence of abrupt phase transition by showing domain formation via time lapse microscopy prior to supersaturation. Furthermore, TRK-T3 condensates formed in vitro recover upon photobleaching (Supp. Fig. 3 E). Together, this supports that the assemblies observed are not aggregates but instead have several hallmarks of a liquid-like phase.

We greatly appreciate Reviewer 2's constructive suggestions as they will be valuable to guide our future work to build on this system *in vitro*.

Reviewer #3 (Comments to the Authors (Required)):

This study examines how the oncogenic fusion protein TRK-T3 activates MAPK signalling by forming planar, sheet-like condensates in the cytosol, distinct from the spherical condensates formed by TFG alone.

The study shows that the condensate geometry is driven by a hydrophobic transmembrane domain (TMD) derived from TrkA. Deletion of the TMD disrupts planar condensate formation and significantly reduces recruitment of GRB2 and downstream ERK phosphorylation. While other domains contribute to signaling, the TMD is the primary determinant of condensate architecture and signaling strength. The data suggest that planar condensates spatially organize kinase domains to promote efficient autophosphorylation, functionally mimicking ligand-induced RTK clustering. A minimal hydrophobic peptide corresponding to the TMD disrupts condensate formation and attenuates signaling, highlighting hydrophobic interactions as a potential therapeutic target.

The paper has major shortcomings which are listed below, the work is very preliminary and needs many validations and would be unsuitable to publish if it cannot answer the following concerns

2. Main points of the paper and major concerns

2.1 TRK-T3 form planar, sheet-like condensate

Concern 2.1.a- What is the endogeneous concentration of TRK-T3 in cells? Do the endogenous proteins form similar condensates ?

We thank the reviewer for these questions. We are unable to obtain patient cell lines to address this concern directly. Furthermore, TRK-T3 is not found as an endogenous protein, as it is formed by a chromosomal translocation, causing a fusion between the N-terminal portion of TFG and the C-terminal portion of the kinase TrkA. We could assume that the expression level of the fusion protein is mediated by the same promoter that controls TFG, resulting in similar expression levels. However, in cancer cells relative expression levels are difficult to assess because of the genomic instability generated by the new fusion. The expression level may be similar to the expression level of TFG, which has endogenous expression levels that are sufficient to drive phase separation of TFG itself (Bogus et al. 2025). Nonetheless, to address the concern, we included new data in Supplemental Figure 1 H and I, where we examine the relative expression of TRK-T3 compared to TFG that is endogenously tagged in HeLa cells (Supp. Fig 1 H). We find that transfection of TRK-T3 as conducted throughout the manuscript results in protein levels 4-8X higher than endogenous TFG on average, and importantly, when examining low-expressing cells via fluorescence microscopy, we find evidence that such cells are also capable of forming TRK-T3 condensates (Supp. Fig 1 I).

Concern 2.1.b-Localisation of the condensates is not very clear. The authors should conduct additional confirmatory experiments to demonstrate that these condensates form on the inner side of the membrane. E.g. if the membrane is disturbed, will the condensates also get disturbed ?

To address the reviewer's concern, we have added 3D reconstructions of TRK-T3 sheets localized adjacent to the plasma membrane (updated Figure 2 A, bottom panel).

Concern 2.1.c- In vitro condensate assays shown in Figure 3D are based on the progressive drying method. This method leads to an increase in concentration of the protein and salt as the water evaporates. This is a very poor experiment to show phase separation as we do not know the final concentration and salt concentration at which the phase separation takes place.

The interactions of TRK-T3 molecules are preserved even in supersaturation with an extreme chemical milieu because the interactions are driven by the features encoded in the protein. This method has been validated by others as a screening method to identify condensation propensity (PMID: 31833055). Similar to reviewer 2's concern (point 4), to distinguish TRK-T3 condensation from aggregation, and to avoid the extremes of supersaturation, we performed timelapse microscopy of recombinant TRK-T3 and observe abrupt phase transitions resulting in liquid-like TRK-T3 condensates in Figure 3 D.

Concern 2.1.4- The material properties of the in vitro condesate is unclear as no FRAp experiments are shown.

We find qualitative evidence that the condensates formed *in vitro* recover upon photobleaching (Supp. Fig 3 E).

2.2 TMD is necessary for the planar geometry of condensates

As depicted in Figure 3 B, 3 C, 3 D, and 3 E, the TrkA TMD that is part of the TRK-T3 fusion protein indeed controls condensate geometry both in cells and *in vitro*.

2.3 TRK-T3 condensates localize near the plasma membrane The authors showed that the TRK-T3 forms planar sheet like condesate, and suggest that these condesates are formed on the underside of the plasma membrane.

Concern 2.3.a- Its not really clear if they are really on the underside of the membrane

To address the reviewer's concern, we have added 3D reconstructions of TRK-T3 sheets localized adjacent to the plasma membrane (updated Figure 2 A, bottom panel).

Concern 2.3.b- FRET experiments should be shown between membrane inner side protein and TRK-T3

While an intriguing suggestion, we do not believe that the suggested experiment will add to the conclusion of our paper, which is to show that TRK-T3 condensation is driven by a short hydrophobic segment and that eliminating this segment results in a loss of condensate geometry, GRB2 recruitment, and MAPK activation.

2.4 Condensate planar geometry is required for the GRB2 recruitment and MAPK activation Since this fusion protein was studied to show how MAPK pathway is activated in cancer, a cellular model system for inducing normal cells to cancerous cells due to this type of fusion protein, dependent on condensate internal geometry would be desirable.

We appreciate the interesting suggestion, but we believe this is not within the scope of the manuscript.

2.5 A minimal TMD peptide can disrupt TRK-T3 condensates Data strongly support a dose-dependent effect of the hydrophobic TrkA-TMD peptide on condensate dissolution, both in cells and in vitro.

Concerns: The Specificity of the TMD peptide has not been tested.

We appreciate that this point has been raised. It will be valuable to establish specificity and devise minimal constructs capable of disruption of the TRK-T3 condensates, as well as small molecule inhibitors, although this is beyond the scope of the current manuscript.

Quantification concerns

Fig. 1. J - no quantification sof mobile fractions or time scales

We have updated Figure 1 J to more clearly show the mobile fraction.

Fig. 3D- No quantification of the fraction of protein in the droplet vs outside

Updated Figure 4 F addresses this concern via measuring of the coefficient of variation of both TRK-T3 and the TRK-T3ΔTMD purified proteins.

Fig. 4B- colocalization coefficients should be calculated, as line profile of 1 foci in 1 cell is cherry picking, and not statistically testable

We have updated Figure 4 B to include the Pearson correlation r value, and we have clarified the text (lines 280 – 281 and 289 – 291).

May 22, 2026

Re: Life Science Alliance manuscript #LSA-2026-03653-TR

Prof. Andreas M Ernst
University of California San Diego
School of Biological Sciences; Cell & Developmental Biology
9500 Gilman Dr
La Jolla 92093

Dear Dr. Ernst,

Thank you for submitting your manuscript entitled "TRK-T3 Condensate Geometry Drives Growth Signaling" to Life Science Alliance.

Your revised manuscript was evaluated by two of the original reviewers. As you will read, both of them acknowledge the added experiments in response to their concerns. However, Reviewer 1 has a few pending concerns. We agree that experimental data to address these might be challenging, however you must resolve them with appropriate edits to your manuscript. We hope it will not take you much time to make these minor edits:

-refrain from overuse of the term 'geometry' in describing the appearance of the condensates, and carefully consider its use given the lack of data supporting condensate shape. This would also apply to the abstract and title of your manuscript.
-acknowledge the confounding data on ER localisation/condensate formation of WT TRK-T3(Figure 2, 4) versus condensate formation ability in ΔTMD (Figure 3) and change in ER localisation. You must acknowledge the mechanism offered by this reviewer and amend the abstract/discussion accordingly.
-detailed description of Figure 5B and as requested in methods.

Overall, we would be happy to publish your paper in Life Science Alliance pending resolution of the reviewer's requests and final changes (below) necessary to meet our formatting guidelines.

-Kindly provide antibody concentrations used for various approaches.
-Kindly expand on details for (1) KDEL-Halo use, (2) excitation/emission filters for co-localisation experiments, (3) calculation of co-localisation coefficients, (4) time-lapse imaging including temperature during image acquisition.
-Please expand on the statement in line 597, ".....via CRISPR/Cas9, and validated by PCR, Sanger sequencing, Western blotting, and fluorescence microscopy." Please provide further details here (e.g. PCR primers, antibodies)
-Please upload your main and supplementary figures as single files and remove them from the manuscript file
-Please upload your main manuscript text as an editable doc file.
-Please add the X and Bluesky handles of your host institute/organisation, as well as your own, and/or one of the authors, in our system
-Please be sure that the authorship listing and order are correct and match between the system and the manuscript file
-Please consult our manuscript preparation guidelines https://www.life-science-alliance.org/manuscript-prep and make sure your manuscript sections are in the correct order
-Please rename "Ethics declarations" to "Conflict of Interest."
-Please add your main and supplementary figure legends to the main manuscript text after the references section.
-It is recommended to exclude figures from the manuscript text and leave them uploaded separately
-There is a callout for the figure S4D, and this figure doesn't have panel D...please correct
-Please add callouts for Figures S1E; S2C; S3D; S4C and S5C-D to your main manuscript text. We also note that you have not referred to the sub-panels (Figure 2A) and request you to refer to the volumetric description in the results.
-Please include a running title that differs from the potentially longer main title.

I would be happy to discuss the revision in more detail via email or phone/videoconferencing. Please let me know which option you prefer, if any.

To upload the revised version of your manuscript, please log in to your account: https://lsa.msubmit.net/cgi-bin/main.plex

You will be guided to complete the submission of your revised manuscript and to fill in all necessary information. Please get in touch in case you do not know or remember your login name.

The typical timeframe for revisions is three months. Please note that papers are generally considered through only one revision cycle, so strong support from the referees on the revised version is needed for acceptance.

While you are revising your manuscript, please also attend to the below editorial points to help expedite the publication of your manuscript. Please direct any editorial questions to the journal office. When submitting the revision, please include a letter addressing the reviewers' comments point by point.

Thank you for this interesting contribution to Life Science Alliance. We hope that the comments below will prove constructive as your work progresses, and we are looking forward to receiving your revised manuscript.

Sincerely,

Sarita Hebbar, PhD
Scientific Editor
Life Science Alliance
http://www.lsajournal.org

A. THESE ITEMS ARE REQUIRED FOR REVISIONS

-- A letter addressing the reviewers' comments point by point.

-- An editable version of the final text (.DOC or .DOCX) is needed for copyediting (no PDFs).

-- High-resolution figure, supplementary figure and video files uploaded as individual files: See our detailed guidelines for preparing your production-ready images, https://www.life-science-alliance.org/authors

-- Summary blurb (enter in submission system): A short text summarizing in a single sentence the study (max. 200 characters including spaces). This text is used in conjunction with the titles of papers, hence should be informative and complementary to the title and running title. It should describe the context and significance of the findings for a general readership; it should be written in the present tense and refer to the work in the third person. Author names should not be mentioned.

-- By submitting a revision, you attest that you are aware of our payment policies found here: https://www.life-science-alliance.org/copyright-license-fee

B. MANUSCRIPT ORGANIZATION AND FORMATTING:

Full guidelines are available on our Instructions for Authors page, https://www.life-science-alliance.org/authors

We encourage our authors to provide original source data, particularly uncropped/-processed electrophoretic blots and spreadsheets for the main figures of the manuscript. If you would like to add source data, we would welcome one PDF/Excel-file per figure for this information. These files will be linked as supplementary "Source Data" files.

IMPORTANT: It is Life Science Alliance policy that if requested, original data images must be made available. Failure to provide original images upon request will result in unavoidable delays in publication. Please ensure that you have access to all original microscopy and blot data images before submitting your revision.

Reviewer #1 (Comments to the Authors (Required)):

The authors have addressed some of my previous concerns through their responses and additional experiments. However, their claim about the geometry of condensates lacks experimental support and is overemphasized. Moreover, the proposed role of the transmembrane segment is unconvincing. These issues need to be resolved before publication.
Major comments:
In Figs. 3B, 4B, and 4C, the authors show that deletion of the TMD (ΔTMD) abolishes TRK-T3 condensate formation and reduces downstream signaling. The authors attribute this to a planar geometry mediated by the TMD that facilitates efficient kinase autophosphorylation. However, this explanation does not fully capture the underlying mechanism. Based on Fig. 2C, TRK-T3 is almost completely colocalized with the ER, and this localization depends entirely on membrane insertion mediated by the TMD. Compared with three-dimensional phase separation, two-dimensional phase separation on membranes requires a lower concentration threshold. It seems that the oligomerization capacity of TRK-T3 is sufficient to drive phase separation on the ER membrane (2D), but insufficient to support phase separation in the cytosol (3D). Therefore, ΔTMD not only loses ER localization, but also loses the ability to form condensates in the cytoplasm. Similarly, wild-type TRK-T3 can form condensates on the ER membrane to efficiently recruit signaling proteins such as GRB2 (Fig. 4B), whereas loss of ER localization restricts it to forming only oligomers in the cytosol, which are insufficient to sustain condensates and thus result in reduced downstream signaling. I suggest the authors consider this mechanism and revise their conclusions accordingly.

Minor comments:
1. In Fig. 5B, the authors should indicate which cells are considered condensate-positive and condensate-negative in the fluorescence images.
2. The sgRNA sequence used for TFG knock-in should be provided in the Methods section.

Reviewer #2 (Comments to the Authors (Required)):

The authors carefully revised the manuscript and, in principle, addressed the concerns raised. Thus. I recommend publishing of this study in Life Science Alliance.

We thank the reviewers the editor for their constructive feedback on our manuscript.

Thank you for submitting your manuscript entitled "TRK-T3 Condensate Geometry Drives Growth Signaling" to Life Science Alliance.

Your revised manuscript was evaluated by two of the original reviewers. As you will read, both of them acknowledge the added experiments in response to their concerns. However, Reviewer 1 has a few pending concerns. We agree that experimental data to address these might be challenging, however you must resolve them with appropriate edits to your manuscript. We hope it will not take you much time to make these minor edits:

- refrain from overuse of the term 'geometry' in describing the appearance of the condensates, and carefully consider its use given the lack of data supporting condensate shape. This would also apply to the abstract and title of your manuscript.

We have addressed this point and greatly reduced our use of the term ‘geometry’ to describe condensate morphology.

- acknowledge the confounding data on ER localisation/condensate formation of WT TRK-T3(Figure 2, 4) versus condensate formation ability in ΔTMD (Figure 3) and change in ER localisation. You must acknowledge the mechanism offered by this reviewer and amend the abstract/discussion accordingly.

We have addressed this point and updated the abstract and discussion accordingly.

- detailed description of Figure 5B and as requested in methods

This point has been addressed.

- Kindly provide antibody concentrations used for various approaches.

Added to Materials and Methods section.

Kindly expand on details for (1) KDEL-Halo use, (2) excitation/emission filters for co-localisation experiments, (3) calculation of co-localisation coefficients, (4) time-lapse imaging including temperature during image acquisition.

Added to Materials and Methods section.

Please expand on the statement in line 597, ".....via CRISPR/Cas9, and validated by PCR, Sanger sequencing, Western blotting, and fluorescence microscopy." Please provide further details here (e.g. PCR primers, antibodies)

Materials and Methods section has been updated.

Please upload your main and supplementary figures as single files and remove them from the manuscript file

Done.

Please upload your main manuscript text as an editable doc file.

Done.

Please add the X and Bluesky handles of your host institute/organisation, as well as your own, and/or one of the authors, in our system

UC San Diego Biological Sciences Twitter: @ucsdbiosciences

UC San Diego Bluesky: @ucsandiego.bsky.social

UC San Diego Twitter: @UCSanDiego

Please be sure that the authorship listing and order are correct and match between the system and the manuscript file

Done.

Please consult our manuscript preparation guidelines https://www.life-science-alliance.org/manuscript-prep and make sure your manuscript sections are in the correct order

Done.

Please rename "Ethics declarations" to "Conflict of Interest."

Done.

Please add your main and supplementary figure legends to the main manuscript text after the references section.

Done

It is recommended to exclude figures from the manuscript text and leave them uploaded separately

Done

There is a callout for the figure S4D, and this figure doesn't have panel D...please correct

Corrected

Please add callouts for Figures S1E; S2C; S3D; S4C and S5C-D to your main manuscript text. We also note that you have not referred to the sub-panels (Figure 2A) and request you to refer to the volumetric description in the results.

Callouts have been added where appropriate.

Please include a running title that differs from the potentially longer main title.

TRK-T3 Phase Separation.

I would be happy to discuss the revision in more detail via email or phone/videoconferencing. Please let me know which option you prefer, if any.

To upload the revised version of your manuscript, please log in to your account: https://lsa.msubmit.net/cgi-bin/main.plex

You will be guided to complete the submission of your revised manuscript and to fill in all necessary information. Please get in touch in case you do not know or remember your login name.

The typical timeframe for revisions is three months. Please note that papers are generally considered through only one revision cycle, so strong support from the referees on the revised version is needed for acceptance.

While you are revising your manuscript, please also attend to the below editorial points to help expedite the publication of your manuscript. Please direct any editorial questions to the journal office. When submitting the revision, please include a letter addressing the reviewers' comments point by point.

Thank you for this interesting contribution to Life Science Alliance. We hope that the comments below will prove constructive as your work progresses, and we are looking forward to receiving your revised manuscript.

Sincerely,

Sarita Hebbar, PhD

Scientific Editor

Life Science Alliance

http://www.lsajournal.org

Reviewer #1 (Comments to the Authors (Required)):

The authors have addressed some of my previous concerns through their responses and additional experiments. However, their claim about the geometry of condensates lacks experimental support and is overemphasized. Moreover, the proposed role of the transmembrane segment is unconvincing. These issues need to be resolved before publication.

We thank the reviewer for raising these concerns, which we address below. We have modified the language throughout the manuscript to deemphasize our claim of planar condensate geometry, and the modified text is colored magenta. We have also addressed the concern regarding the proposed role of the TMD in the discussion.

Major comments:

In Figs. 3B, 4B, and 4C, the authors show that deletion of the TMD (ΔTMD) abolishes TRK-T3 condensate formation and reduces downstream signaling. The authors attribute this to a planar geometry mediated by the TMD that facilitates efficient kinase autophosphorylation. However, this explanation does not fully capture the underlying mechanism. Based on Fig. 2C, TRK-T3 is almost

completely colocalized with the ER, and this localization depends entirely on membrane insertion mediated by the TMD. Compared with three-dimensional phase separation, two-dimensional phase separation on membranes requires a lower concentration threshold. It seems that the oligomerization capacity of TRK-T3 is sufficient to drive phase separation on the ER membrane (2D), but insufficient to support phase separation in the cytosol (3D). Therefore, ΔTMD not only loses ER localization, but also loses the ability to form condensates in the cytoplasm. Similarly, wild-type TRK-T3 can form condensates on the ER membrane to efficiently recruit signaling proteins such as GRB2 (Fig. 4B), whereas loss of ER localization restricts it to forming only oligomers in the cytosol, which are insufficient to sustain condensates and thus result in reduced downstream signaling. I suggest the authors consider this mechanism and revise their conclusions accordingly.

We respectfully disagree that TRK-T3 colocalization with the ER depends on membrane insertion mediated by the TMD, as there is signal sequence to promote insertion as for native RTKs. TRK-T3 has been extensively characterized and established as a cytosolic fusion protein (Greco et al 1995, Greco et al 1998, Witte et al 2011). However, we cannot discount that TRK-T3 condensates are templated by association with the ER (as peripheral membrane proteins) and have revised the text to reflect the possibility of this mechanism more clearly (lines 401 – 403).

Minor comments:

1. In Fig. 5B, the authors should indicate which cells are considered condensate-positive and condensate-negative in the fluorescence images.

We thank the reviewer for this suggestion and have updated Fig. 5B.

2. The sgRNA sequence used for TFG knock-in should be provided in the Methods section.

We have updated the Methods section by describing the validation performed both by the company who created the knock-in (ExpressCells) as well as our own validation via WB and fluorescence microscopy.

Reviewer #2 (Comments to the Authors (Required)):

The authors carefully revised the manuscript and, in principle, addressed the concerns raised. Thus. I recommend publishing of this study in Life Science Alliance.

We thank Reviewer 2 for their support of our work and thoughtful and constructive feedback during prior revisions.

June 5, 2026

RE: Life Science Alliance Manuscript #LSA-2026-03653-TRR

Prof. Andreas M Ernst
University of California San Diego
School of Biological Sciences; Cell & Developmental Biology
9500 Gilman Dr
La Jolla 92093

Dear Dr. Ernst,

Thank you for submitting your Research Article entitled "TRK-T3 Condensate Organization Drives Growth Signaling". It is a pleasure to let you know that your manuscript is now accepted for publication in Life Science Alliance. Congratulations on this interesting work.

The final published version of your manuscript will be deposited by us to PubMed Central upon publication.

Your manuscript will now progress through copyediting and proofing. It is journal policy that authors provide original data upon request.

Reviews, decision letters, and point-by-point responses associated with peer-review at Life Science Alliance will be published, alongside the manuscript. If you do want to opt out of having the reviewer reports and your point-by-point responses displayed, please let us know immediately.

IMPORTANT: If you will be unreachable at any time, please provide us with the email address of an alternate author. Failure to respond to routine queries may lead to unavoidable delays in publication.

Scheduling details will be available from our production department. You will receive proofs shortly before the publication date. Only essential corrections can be made at the proof stage so if there are any minor final changes you wish to make to the manuscript, please let the journal office know now.

DISTRIBUTION OF MATERIALS:
Authors are required to distribute freely any materials used in experiments published in Life Science Alliance. Authors are encouraged to deposit materials used in their studies to the appropriate repositories for distribution to researchers.

You can contact the journal office with any questions, contact@life-science-alliance.org

Again, congratulations on a very nice paper. I hope you found the review process to be constructive and are pleased with how the manuscript was handled editorially. We look forward to future exciting submissions from your lab.

Sincerely,

Sarita Hebbar, PhD
Scientific Editor
Life Science Alliance
http://www.lsajournal.org
